# Healthcare providers’ perspectives of barriers and facilitators to highly active antiretroviral therapy adherence among HIV-positive women in Southern Ethiopia: A qualitative study

**DOI:** 10.1371/journal.pone.0312980

**Published:** 2025-09-19

**Authors:** Alemayehu Abebe Demissie, Elsie Janse van Rensburg

**Affiliations:** 1 Department of Health Studies, University of South Africa (UNISA), Ethiopia Campus, Addis Ababa, Ethiopia; 2 Hawassa University College of Medicine and Health Sciences, Hospital Public Health, Hawassa, Ethiopia; 3 Department of Health Studies, University of South Africa (UNISA), Pretoria, South Africa; University of Ottawa, CANADA

## Abstract

**Background:**

Following a strict regimen of highly active antiretroviral therapy (HAART) is the fundamental factor in achieving successful treatment outcomes for HIV/AIDS. Poor adherence to HAART not only amplifies the risk of HIV transmission but also leads to deteriorating health, treatment failures, and a rise in drug-resistant HIV strains, ultimately contributing to increased morbidity and mortality rates. Therefore, the objective of the study is to explore and describe the perceptions of healthcare providers about HAART adherence of women in Southern Ethiopia who are HIV positive.

**Methods:**

Focus group discussions (FGDs) were used among 27 healthcare providers (HCPs) (13 medical doctors and 14 nurses) to determine the extent of HAART adherence among HIV-positive women in Southern Ethiopia. The interviews were conducted in the local Amharic language and were audio recorded with permission from the participants. The FGDs were transcribed verbatim, coded for themes, categories and subcategories, and analyzed using thematic data analysis.

**Results:**

The findings of the study reflected two themes: barriers to and facilitators of HAART medication adherence among HIV-positive women. These included patient-related factors, treatment-related factors, psychosocial-related factors, family- and community-related factors, and healthcare service-related factors. The following were identified as barriers to HAART adherence: Stigma and discrimination, gender roles, lack of money for food and transport, depression, busy schedule, forgetting of doses, religion, drug side-effects, pills burden, and size, dosage frequency, long waiting time, and unavailability of services at weekends. However, perceived benefits of HAART, family responsibility, reminders, family support, dosage formulation, experiencing improved health on HAART, relationship with healthcare providers, adherence counselling and education, and adherence supporting peer groups were identified as facilitators of HAART adherence.

**Conclusions:**

Adherence to HAART medication is a major challenge among HIV-positive women in Southern Ethiopia. Therefore, tailored strategies to enhance HAART medication adherence should be targeted to address the barriers identified in the study.

## Introduction

Globally, the number of individuals with HIV/AIDS receiving antiretroviral therapy (HAART) has increased significantly, rising from 7.7 million in 2010 to 30.7 million in 2023. Despite significant advancements in enhancing access to HAART, merely 68% of individuals undergoing therapy have attained viral suppression [1]. The global rate of viral suppression among HIV patients is 88.4%, indicating that insufficient adherence to HAART poses a significant barrier to HIV/AIDS treatment [2]. Ethiopia is significantly impacted by HIV epidemics, with a notable prevalence of the virus among women. Currently, women account for a majority of new infections and AIDS-related deaths [3]. A total of 610,000 individuals were diagnosed with HIV in Ethiopia, including 27,000 children under 15 years and 513,990 adults and children are receiving HAART in 2023 [4]. Adherence to HAART is essential for achieving optimal virological outcomes and reducing the likelihood of drug resistance, morbidity, and mortality [5]. A systematic review and meta-analysis conducted in Ethiopia found the pooled prevalence of poor HAART adherence to be 20.68%, which is significantly high [6]. A related study carried out in Ethiopia found that merely 73.1% of patients adhering to their treatment were adherent to HAART [7]. Poor HAART adherence arises from a range of factors, including patient and familial circumstances, economic situations, medication-related obstacles, and the status of healthcare providers and systems. Studies reveal that women face significant challenges in adhering to HAART, particularly related to education, age, poverty, stigma, and socio-familial support [8]. In addition to their other roles, women manage household responsibilities, which can create additional challenges in balancing their family and social lives [9]. There is a lack of studies conducted on the adherence of HIV-positive women to HAART in Ethiopia specifically. To address this gap, organizations, and care providers need to come up with a combination of practical interventions to maximize the adherence of women to HAART based on global and Ethiopian contexts. In light of related research done in Ethiopia, this study aims to address the adherence to HAART among HIV-positive women from a unique perspective by focusing on healthcare providers. While previous studies have explored the barriers and facilitators of adherence from the patients’ viewpoints, this investigation seeks to complement existing literature by examining how healthcare providers perceive and influence adherence behaviors. This approach not only enriches the understanding of adherence dynamics but also provides valuable insights that can inform interventions aimed at improving treatment outcomes for HIV-positive women in South Ethiopia.

As a result, this study attempts to bring about a more comprehensive understanding of the adherence of HIV-positive women to antiretroviral treatment in Southern Ethiopia with the goal of overcoming the gaps in adherence to HAART medications.

## Materials and methods

### Design and setting

The study used an exploratory qualitative research design to gain new insights and understandings based on the themes, categories, and subcategories that emerged from the data.

The study applied the consolidated criteria for reporting qualitative research (COREQ), which is a set of criteria for reporting qualitative research (see S1 Checklist) [10].

#### Study setting and sampling procedures.

The researchers used a purposive and convenient sampling procedure to select the Southern Ethiopian region and the three urban hospitals that were studied. This sampling technique was chosen to select the Southern region from the nine regions of Ethiopia and the study hospitals from 21 urban public hospitals found in Southern Ethiopia based on convenience (most accessible to and easily reached by the researchers) and a load of HIV-positive individuals. The purpose of the selection of these three urban public hospitals was due to the high volume (load) of HIV-positive individuals who were taking HAART compared to those in other health facilities in Southern Ethiopia.

Healthcare providers (HCPs) working at the HAART clinics of the study hospitals were purposively and conveniently sampled based on the eligibility criteria. Three FGDs with twenty-seven participants (13 medical doctors and 14 nurses) were purposively and conveniently sampled for the FGDs. Participant distribution across the FGDs was ten, nine, and eight in each respective session.

#### Eligibility criteria.

Participants were eligible if they were healthcare providers (HCPs) in Southern Ethiopia, working as either a nurse or doctor in the HAART clinic of one of the sampled study hospitals for at least one year, and were willing and available to participate in a focus group.

#### Focus group discussion guide development and data collection procedure.

A semi-structured FGD guide was developed based on the literature reviewed. The guide was designed to explore and describe the perceptions of healthcare providers regarding HAART adherence among HIV-positive women in Southern Ethiopia (see S1 Data). Focus group discussions were chosen as the primary data collection method for this study due to the sensitivity of the research topic, as this approach fosters a supportive environment that enhances confidentiality and encourages open dialogue among participants. To enhance the credibility of the discussion guides, one FGD was conducted as a pre-test before the actual data collection commenced.

The intended data were collected from healthcare providers (HCPs) working at HAART clinics in the study hospitals located in Southern Ethiopia. The FGDs were guided by the discussion guide, allowing for probing questions to be posed when additional insights were necessary [11]. The focus group discussions were held until data saturation was achieved, that is, where no more themes, concepts and insights would be acquired through subsequent discussions [12]. Field notes were taken to capture behaviors, impressions, nonverbal actions, and contextual elements that may not have been fully recorded through audio during the discussions.

This methodology facilitated rich interactions among participants, enabling them to share their insights and experiences collaboratively. The FGDs provided a comprehensive understanding of the challenges and perspectives related to HAART adherence from the viewpoint of healthcare providers, contributing valuable qualitative data to the research.

### Data collection and analysis

We conducted three FGDs with 27 HCPs. The FGDs were audio-recorded (with the participant’s permission). The discussions were conducted in Amharic, a local language that most people in Ethiopia speak. The FGDS lasted approximately 90–120 minutes. The researchers used field notes in the in-depth interviews to substantiate the findings from this qualitative data-gathering technique [13]. The data were collected in June 2023. The researchers translated the audio-recorded data from Amharic into English for analysis. The data from FGDs and field notes were analyzed using the thematic analysis method, which involves searching across a dataset (discussions, field notes, or a range of data) to find repeated patterns of meaning. The researchers used the Nvivo 12 qualitative data analysis computer program for data analysis [14]. The researchers used thematic analysis for the current study since it is recommended as a convenient method for employing a participatory research paradigm with participants as collaborators [15]. Finally, some verbatim quotes from HCPs were used to illustrate the subcategories.

### Reflexivity

As part of the important practice of reflexivity, which is essential in qualitative research, the authors acknowledge that as one of the authors is a healthcare provider, he may have had biases regarding the experiences of the individual participants.

### Ethical considerations, rigor, and trustworthiness

This study obtained scientific approval from the scientific committee (Department of Health Studies and ethical clearance (Ref: HSHDC/463/2015)) of the College Research Ethics Committee of the University of South Africa (UNISA). Similarly, permission to carry out the study was obtained from the stakeholders, including the Southern Regional Health Bureau and the three public hospitals where the study was conducted. Confidentiality was ensured at all stages of the process. Informed consent was obtained from the participants prior to the interviews. Participants provided written informed consent before enrollment in the study. Participants were assured that refusal to participate or withdrawal from the study would not affect their access to healthcare services at the clinic. The researchers considered the essential values of ethical research, which incorporate respect for human autonomy and dignity, justice, beneficence, and nonmaleficence [16]. To ensure the reliability and validity of the results, different techniques were applied. These included developing a rapport with participants, creating a coding system, implementing peer review for themes and subthemes, triangulating data sources (including FGDs transcripts and field notes), and providing a thorough contextualized data description [17–19].

## Results

### Socio-demographic characteristics of study participants

A total of twenty-seven healthcare providers, including medical doctors and nurses, who worked in the HAART clinics of the study urban hospitals have participated in the FGDs. Of the participant healthcare providers, 13 of them were medical doctors and the remaining 14 were nurses. Most healthcare providers participating in the focus group discussions were female (14/27, 51.9%), and the remaining (13/27, 48.1%) were male. Table 1 provides the demographic and background characteristics of the healthcare providers involved in the focus group discussions.

**Table 1:** Summary of demographic characteristics of the HCPs who participated in FGDs.

Characteristics (N = 27)	Profession		Total
	Medical doctor	Nurse	
**Gender**			
Male	8 (61.5%)	5 (35.7%)	13 (100%)
Female	5 (38.5%)	9 (64.3%)	14 (100%)
**Age (yrs.)**			
20–29	5	6	40.7%
30–39	4	5	33.3%
40–49	4	3	25.9%
**Years of Experience in HIV treatment**			
1–5 Years	6 (46.2%)	6 (42.9%)	(100%)
6–10 Years	4 (30.7%)	5 (35.7%)	(100%)
>10 Years	3 (23.1%)	3 (21.4%)	(100%)

### Themes, categories, and subcategories

The perception of HCPs about HIV-positive women’s HAART adherence was described in two themes. These themes included barriers to HAART medication adherence and facilitators of HAART medication adherence. Barriers to HAART medication adherence among HIV-positive women were grouped into three categories and twelve subcategories (Table 2). Facilitators of HAART medication adherence were grouped into three categories and nine subcategories (Table 3).

**Table 2:** Barriers to HAART medication adherence among HIV-positive women in Southern Ethiopia.

Theme	Categories	Subcategories	Selected HCPs quotes
Barriers to HAART adherence	Patient-related factors	Stigma and discrimination	• “To avoid their identification by others in their residential area, some HIV-positive women prefer to follow their treatment [HAART] at another hospital like from Yirgalem [a town about 42 km away] when they get access to it in Hawassa [a town where one study hospital is], and vice versa. After some time, they will be challenged [lack of transportation, distance etc.] while they try to re-fill their medications during the scheduled day. Patients in such circumstances will not attend their treatment properly.” (FGD2-P5). • “During their visit to our clinic, many HIV-positive women tell us about their fear of identification of their HIV status. In my observation, the stigma will occur, especially in female patients by their family members, friends and other people at their workplace. One woman patient, who is a mother of three children, told me that she had stopped socializing with her neighbours due to their reactions to her children having HIV.” (FGD3-P8).
		Gender role	• “... therefore, women in our community are expected to spend their time more in their homes and villages by taking care of their children, partners, and family members and by doing activities around their homes and village. Due to that, those HIV-positive women may not have a chance to visit even the nearby healthcare facility for their illnesses and treatment. Therefore, this gender-based cultural norm implies [affects] HAART adherence of those HIV-positive women taking HAART.” (FGD1-P4). • “I know an HIV-positive woman in my encounter who has two children with her. She is a single mother; she always takes her children to school early in the morning and brings them back to their home late afternoon. She always provides care for them and does everything around the home. She sometimes couldn’t take her pills. It is very challenging for HIV-positive women to take their HAART regularly as it has been prescribed because of their multiple roles in their family and community.” (FGD1-P3).
		Lack of money for food and transport	• “We hear from some HIV–positive women that they are not able to be hired by the organisation or private homeowners because of their HIV-positive status, and this unemployment makes patients unable to get access to food and this also enforces them to travel too far just to seek any work opportunity. Finally, these women don’t get money for their daily expenses and their transportation costs to the hospital.” (FGD2-P10). • “Some patients tell us that they have no money as most of them are not employed because they are not educated well. If they get a job they would be working at a low wage rate and working for long hours. Due to that, they can’t afford the expenses for food and transportation to come to the HAART clinic.” (FGD1-P2).
		Depression	• “Patients tell us that they are not interested to take medications at the right time when they go through a depressive mood. This is one of the reasons for some patients [to] miss or don’t take their medications properly.” (FGD2-P3). • “We have observed that those HIV-positive women who had been diagnosed with some kind of psychiatric problems, for example, depression; usually don’t take their medication as prescribed by the healthcare providers because of the depressive mood they have.” (FGD3-P7).
		Busy schedule	• “These HIV-positive women are also busy with their social activities in their village, for example, they may participate in Idir [a kind of social gathering] gatherings during the death of community members. Therefore, they are sometimes busy and vulnerable to missing their dose as we have seen in their life experience.” (FGD1-P10). • “They do not have enough time to take their medication as they are responsible and busy with multiple gender-based responsibilities.” (FGD2-P8).
		Forgetting doses	• “She told me that she went to another town which is almost 275 km away from her residence to attend a funeral ceremony as her best friend’s father had died. As she was in a hurry, she forgot to take her ARV medication with her and she stayed there for five days without taking her pills.” (FGD1–5). • “Travel to somewhere too far from their residential area, is one of our patients’ reasons for forgetting their pills to take. When they travel, they usually forget to take their pills with them and thus, until they return to their residence, they wouldn’t take their pills.” (FGD2-P9).
			• “Before one year, one HIV-positive woman who follows her HIV treatment in our clinic told me that after a week’s prayer for her, the pastor told her that she had been cured and thus he ordered he ordered her to throw her medication [away] forever.” (FGD 1-P5).
		Religion	• “Some HIV-positive women patients come to the clinic after they have lost to follow-up their HAART for months. They do not come to the clinic [HAART clinic] on their scheduled date for refilling their medications. When we ask them why they don’t attend the clinic for their medication refilling during their scheduled date, they tell us their reason as they were in a monastery for holy water.” (FGD2-P2).
	Treatment-relatedfactors	Drug side-effects	• “There are patients who strongly complain that HAART has more side defects in women than men as compared to its benefit. They [some women] tell you that their body shape has been changed badly due to the ARVs they are taking.” (FGD3-P9). • “I “Another major influencing factor for HIV-positive women not to take their medication properly is the drug side effect.” (FGD1-P5).
		Pills burden and size	• “The “Yes, some patients complain about the burden they have due to the pills taken daily for a long time. Due to that, they tell us that they have a load from a daily long time taking pills and thus, they sometimes miss their doses.” (FGD1-P10). • “In addition to that, they also tell us the size of ARVs is big to swallow and creates inconvenience in women during the taking of their medication. Due to this, some women skip some doses just to avoid and forget the inconvenience which would be created by the big size of ARVs.” (FGD2-P2).
		Dosage frequency	• “Sometimes, they tell you that it is challenging for them to take ARVs two times every day of their life, especially when taking these pills with other kinds of pills and the daily intake that is required to be coupled with other additional drugs for other illnesses.” (FGD3-P2). • “It is clear that taking many pills two times a day, especially for a long time, is challenging for our patients; it is one of the reasons for [why] patients have poor HAART adherence.” (FGD3-P8).
	Healthcare services-related factors	Long waiting time	• “There are times that patients need to have investigations, for example, laboratory, psychiatric, and gynaecological, when they come to the clinic on the scheduled date to collect their medication and thus they may stay long hours in the hospital. However, some patients don’t stay here long and leave the hospital without refilling their medication.” (FGD2-P8). • “There are many challenges we identified through the assessment of adherence to HAART in HIV-positive women... long waiting times at clinics due to different reasons, and lack of services at weekends are some of the challenges I have seen.” (FGD2-P1).
		Un availability of services at weekends	• HAART service provision is only given to patients from Monday through Friday but due to the nature of their work, there are some HIV-positive women who don’t have free time during these days and thus, they may not come to the clinic and collect their medication timely.” (FGD2-P1).
			• “There are patients who tell us that the scheduled date given by the clinic to refill their medications is not comfortable to them because they are labourers, and have no time from Monday through Saturday unless they have been given appointments on Sunday [work free day]. I remember two women who skipped on repeatedly their scheduled date to refill their ARV medications due to being unable to come to the clinic during weekdays.” (FGD3-P9).

**Table 3:** Facilitators of HAART medication adherence among HIV-positive women in Southern Ethiopia.

Theme	Categories	Subcategories	Selected HCPs quotes
Facilitators of HAART adherence	Patient-related factors	Perceived benefits of HAART	• “Many HIV-positive women have better knowledge and understanding of the benefits of HAART and good adherence. These patients collect their medications from the HAART clinic on time and take them appropriately on a daily basis.” (FGD2-P1). • “I know a couple, namely a husband and wife who always collect their medication on the clinic’s scheduled date and take their medication properly. The wife told me that they have 2 children and that she takes care of them, she now has a good health condition because of HAART and thus HAART means life for her.” (FGD3-P4).
		Family responsibility	• “What I have understood in my day-to-day work in the HAART clinic is, that there are very careful HIV-positive women taking their medication because they want to live longer without ill health due to HIV as they want to provide care for their children, husbands and other family members; unless they are healthy they wouldn’t be responsible to their family.” (FGD1–5). • “I know who are single parents, namely, single moms of children because their partners have died, they have been taking their medication daily at the right time to make themselves healthy, to work hard just to get money for their children’s growth.” (FGD1-P3).
		Reminders	• “We always advise our patients to have a reminder if they are at work which might make them busy and forget to take their medications. Some patients tell us that they are using either a watch or cell phone alarm to remind them to take their medications on time.” (FGD2-P9). • “Currently, many HIV-positive women use medication-taking reminders other than any supporting person. Therefore, these women use the alarms of their cell phones and watch as a reminder method because they want to take their medication appropriately at the right time as prescribed by healthcare providers.” (FGD2-P10).
		Family support	• “Those women who are taking HAART and have family support are taking their medication according to the recommended dose and time.” (FGD2-P7). • “Yes, we have seen that family support is highly important for HIV-positive women, especially for those who are taking their HAART. Because to take their medication at the right time daily, these patients need to have all aspects of support from their close family members. If they get economic and emotional and are reminded of medication-taking support, HIV-positive patients can take their medication properly without fear and discomfort.” (FGD3-P1).
	Treatment-related factors	Dosage formulation	• “These days the drug formulation is attractive and the frequency of taking these drugs is reduced to a single tablet per day. This condition makes women patients take their medication easily and have good adherence to their medication.” (FGD2-P5). • “Some of the recent ARV formulation[s] which contains [contain] three drugs in a fixed tablet is very convenient because our patients [HIV-positive women] tell us they take their daily pill properly since it is single and only once a day.” (FGD1-P10).
		Experiencing improved health on HAART	• “Because many patients [HIV-positive women] have got tested and diagnosed with HIV after they had developed different HIV-related illnesses due to that they know how they had suffered a lot. After they have started taking their medication, their health becomes improved, therefore, they are committed to taking their medication appropriately.” (FGD3-P2). • “Many patients had developed different opportunistic infections (OIs) before they were diagnosed with HIV and even after they knew their HIV-positive status due to fear of starting HAART. But after they have started taking HAART, a significant improvement in health conditions due to HAART makes them stronger and to take their medication properly as prescribed by healthcare providers.” (FGD2-P2).
	Healthcare services-related factors	Relationship with healthcare providers	• “We have a good relationship with our patients. We always try to serve patients with respect and a good approach. That is why they are coming and collecting their medication from the HAART clinic always during their clinic schedule.” (FGD3-P4). • “We always try to serve HIV-positive women as well as men with effective communication and trust. This makes most of these patients have good adherence to their appointments given by our clinic and finally, helps them [HIV-positive women] to take their medication properly as prescribed, which means the right dose at the right time and frequency. “(FGD2-P10).
		Adherence counselling and education	• “Patients tell us that they have changed their negative thoughts about HAART after they have been engaged in adherence and counselling provided by healthcare providers at the HAART clinic.” (FGD1-P1). • “Adherence counselling on HAART medication is really important for our patients not only for new patients and those with poor adherence to HAART but also for those who are on good adherence just to get them motivated in taking their pills and staying in their good adherence. We have observed and understand from our patients that those who have developed a better understanding of the benefits of [and] adherence to HAART through counselling and education have good adherence to their medication.” (FGD3-P2).
		Adherence supporting peer groups	• “They are so important for our patients because these peer-supporting group members are all females and have good adherence to their HAART. They share their life experiences with patients. Always, they also create a mini-discussion forum for patients who are newly initiated [to] HAART to discuss any individual and treatment-related issues.” (FGD1-P1). • “Some women taking ART tell us the support they get from the adherence supporting group helps them take their medication in the right manner.” FGD2-P3.

#### Theme 1: Barriers to HAART adherence.

***Category 1: Patient-related factors.* Stigma and discrimination:** Healthcare providers frequently highlight a common issue, namely, fear of stigma and discrimination faced by HIV-positive women taking HAART in accessing HIV treatment. The fear of stigma and discrimination may lead some HIV-positive women to seek treatment and medical follow-up in other hospitals in other towns, which can cause problems in gaining access to HAART and attending clinic-scheduled appointments. These problems potentially create a substantial influence on HIV-positive women’s ability to adhere to treatment regimens, as well as their overall health and well-being. Health service providers in HAART clinics have often faced this situation.

**Gender role:** Healthcare providers reported that gender-related issues were one of the difficulties affecting HIV-positive women’s ability to take their HAART medication regimen in the right way as prescribed by healthcare providers. They specifically draw attention to how social norms and expectations would restrict the freedom and mobility of HIV-positive women, making it challenging for them to access healthcare services and follow their HIV treatment without the knowledge of their male partners. This may make it difficult and complex for them to take their medications as prescribed, and it may affect how well they adhere to their HAART medications. Thus, gender-related cultural and societal norms could hamper HIV-positive women, further complicating their ability to manage their condition primarily related to their HIV medication adherence.

**Lack of money for food and transport:** Participants (healthcare providers) indicated that the lack of economic sources can affect HIV-positive women’s potential to fill their nutritional needs. Proper nutrition is critical for preserving a robust immune system and supporting the effectiveness of HAART. Without access to nutritious food, HIV-positive women may experience malnutrition, a weakened immune system, and high susceptibility to infections. Because of such reasons, HIV-positive women cannot take their medication correctly according to the prescription.

**Depression:** During the FGDs, healthcare providers reported that depression was one of the barriers affecting HIV-positive women’s ability to take their HAART medication regimen as prescribed by healthcare providers.

Depression negatively influences an HIV-positive individual’s quality of life, social circumstances, HAART adherence, and therapeutic outcomes. It can also increase the likelihood of experiencing other medical and psychiatric issues, unemployment, and disability. Adherence to HAART is considered a dynamic phenomenon that may change over time. It may depend on the mood of HIV-positive individuals and the factors influencing their mood and well-being.

**Busy schedule:** The healthcare workers who participated in this study indicated that HIV-positive individuals sometimes miss taking their HAART medication due to a busy schedule as they may be away from home or attending social gatherings like Ikub and Idir (gatherings of traditional savings associations), funeral ceremonies, or other family and community responsibilities. These responsibilities can make finding enough time for HAART medication intake challenging, leading to missed doses.

**Forgetting doses:** Forgetting the HAART doses was among the issues reported by most healthcare providers as a challenge for optimal HAART adherence. HIV-positive women may have a busy lifestyle that includes travelling somewhere away from their homes, engaging in different social affairs (weddings, funerals, social gatherings, and others), and for personal reasons. Thus, these situations may create mental stress and insufficient time to remember or take their doses correctly, resulting in missed HAART doses.

**Religion:** In the FGDs with the healthcare providers, participants perceived religion to be negatively impacting HIV-positive women’s adherence to HAART. In the view of participants, some patients’ religious beliefs override medical advice, sometimes causing them to miss or discontinue HAART medication. Most healthcare providers revealed that some patients were advised to stop taking HAART after joining some religion because the pastor claimed they would pray for patients to be cured of the disease. Some HIV-positive women stop their medication entirely due to the medically wrong information from religious groups.

***Category 2: Treatment-related factors.* Drug side effects:** In the FGDs, the HCPs noted that many HIV-positive women tell them that HAART is an essential component of HIV treatment due to its effect on improving the immune system, suppressing the virus, and reducing HIV transmission. However, despite recognising its benefit, some HIV-positive women have fears regarding the possible side effects associated with the HAART medication they have been taking. Due to this concern or issue, some HIV-positive women decide to take their medication but skip the pills for some days.

**Pill burden and size:** From the commonly cited factors that interfere with patients’ adherence, the burden and size of pills are some of the factors that affect adherence in the lifelong treatment regimen of HIV/AIDS. According to healthcare providers, though the HIV treatment medication burden is not such a major problem in recent times, some HIV-positive women still complain about the pill burden regarding their medication. The complaint is not only associated with ARVs but also with some other additional drugs taken in parallel.

**Dosage frequency:** The healthcare participants indicated that HIV-positive individuals’ adherence to HAART can be affected by multiple frequency dosing in different ways. Challenges to integrating it into a daily routine, forgetting to take daily medication multiple times a day, and treatment fatigue are some of the potential problems that can occur by increased dosing frequency. Individual preferences, lifestyles, and support networks can all impact adherence. While some people might find it burdensome, others might prefer the structure and routine of taking medication several times per day.

***Category 3: Healthcare services-related factors.* Long waiting time:** Participant healthcare providers pointed out that though they usually try to give the required service on time, some patients do not like to wait at the HAART clinic to refill their medication or attend other medical check-ups. They sometimes leave the hospital without refilling their medication because they are tired of waiting. Long waiting time is, therefore, identified as a barrier to optimal adherence to HAART.

**Unavailability of services at weekends:** Healthcare professional participants indicated that the unavailability of HAART clinic services at weekends may affect HIV-positive individuals’ treatment negatively. This may cause discomfort in these HIV-positive individuals and cause inconsistent clinic attendance and missing HAART regimen doses. Adherence to HAART includes regularly engaging in clinical appointments and taking proper medication as prescribed by healthcare providers. Sometimes, HIV-positive individuals are unable to refill their HAART medications on weekends due to the closures, leading to missed doses.

#### Theme 2: Facilitators of HAART medication adherence.

***Category 1: Patient-related factors.* Perceived Benefits of HAART:** Healthcare providers explained that most HIV-positive women taking HAART and following their treatment at the HAART clinics have a better understanding of the benefits of their medication. Therefore, though these women have experienced some side effects from their medication, they continue taking the pills because of the benefits they get from HAART.

**Family responsibility:** In the FGDs, healthcare providers highlighted that there were HIV-positive single mothers whose partners had died. These mothers have been taking their HAART medication regularly as prescribed by healthcare providers to carry out their familial responsibilities, such as being able to do more in their children’s upbringing and well-being.

**Reminders:** Some healthcare providers in the FGDs highlighted that some HIV-positive women used reminders like a watch or cell phone alarm to remind them of the right time to take their HAART medication. Among the different tools or strategies being used by HIV-positive people are clocks and cell phone alarms, watches, Radios and TVs. A cell phone alarm is a useful medication reminder that many HIV-positive individuals apply to remind them to take their prescribed HAART medication regimen.

**Family support:** Healthcare providers in this study described how lack of family support is a common problem among HIV-positive individuals. Those who do have support from their family are more likely to adhere to their clinic appointments and HAART medication regimens. The support provided by family members can help these HIV-positive individuals have a more comfortable environment to take their prescribed HAART medication.

***Category 2: Treatment-related factors.* Dosage formulation:** Healthcare providers noted that many HIV-positive women were taking simple HAART regimens, namely a combined single tablet, which makes their dose easy to take at the right time. The formulation of the HAART medication regimen and adherence to HAART are interrelated, and their association is complicated and multifaceted. The number of tablets per dose, the frequency of administration, and the complexity of the medication regimen, including side effects, are some of the factors that possibly affect HIV-positive individuals’ adherence to their prescribed medication.

**Experiencing improved health on HAART:** In the FGDs, healthcare providers revealed that many HIV-positive women have already experienced various HIV-related illnesses, which can cause various problems and suffering. These patients started to feel better and saw improvements in their health once they started to take HAART medication. They were encouraged to take their HAART medication properly as they understood the improvement to have resulted from taking HAART medication.

***Category 3: Healthcare services-related factors.* Relationship with healthcare providers:** In the FGD, some healthcare providers explained that they have a strong relationship of trust and good communication with HIV-positive women during their treatment at the HAART clinics. Due to that, these women feel comfortable coming to the HAART clinic during their appointments, and thus, this leads to better adherence to their clinic attendance and HAART medication regimen.

**Adherence counselling and education:** Healthcare providers who participated in the FGD in this research discussed the importance of adherence counselling for HIV-positive individuals who are starting HAART medication or who have been on medication for some time. They emphasized that adherence counselling provided by healthcare providers can help HIV-positive individuals understand the importance of HAART medication and the consequences of poor medication adherence. It also helps HIV-positive individuals address any concerns they have that need medical attention.

**Adherence supporting peer groups:** Healthcare provider participants indicated that the presence of support groups either at the facility or community level is helpful for HIV-positive individuals in taking HAART medication properly. It also creates a comfortable environment for sharing important experiences regarding proper medication taking and social support. The support provided by support groups encourages and boosts the HIV-positive individuals’ self-esteem, thereby increasing adherence to HAART medication.

## Discussion

During the FGDs, the participants shared their valuable views about different barriers they perceived that challenged HIV-positive women’s adherence to their prescribed HAART medication. Based on their experiences and interactions with these HIV-positive women, participants mentioned several key challenges or factors that affect the appropriate taking of HAART regimens. These include stigma and discrimination, gender roles, lack of money for food and transport, depression, busy schedule, forgetting doses, religion, drug side effects, pill burden and size, dosage frequency, long waiting time, and unavailability of services at weekends.

Fear of stigma and discrimination can affect HIV-positive women’s ability to disclose their HIV status to their partners and others. Consequently, this may affect the overall adherence to HAART among HIV-positive women. Most of the healthcare providers from the three study urban public hospitals who participated in the FGDs said stigma and discrimination were still problems for HIV-positive women taking HAART. They mentioned that many HIV-positive women who are receiving their HAART from these hospitals were not free to take their medication in front of others due to the fear of stigma and discrimination if others knew their HIV status. Stigma and discrimination caused HIV-positive individuals trouble in taking, obtaining, and keeping their HIV medications. A study conducted in 2020 in Zambia and South Africa indicated that among the HIV-positive individuals who participated in the study, 25.7% experienced community stigma, 21.5% experienced internalized stigma, and 5.7% a health setting stigma [20]. The study also showed that the probability of stigmatized HIV-positive individuals being non-adherent to their HIV treatment regimen was 1.13 times higher compared to those without stigma and discrimination [21].

The FGD participants also reported that gender-related issues were one of the difficulties affecting HIV-positive women’s ability to take their HAART medication regimen in the right way as prescribed by healthcare providers. They specifically draw attention to how social norms and expectations would restrict the freedom and mobility of HIV-positive women, making it challenging for them to access healthcare services and follow their HIV treatment without the knowledge of their male partners. A study revealed that HAART adherence level is lower in women than in males [22]. These differences in HAART adherence between women and men are related to the discriminatory gender-based duties, responsibilities, and cultural norms that women have in the community and their families. Lack of money for food and transportation was frequently raised as a major concern of HIV-positive women. As reflected by healthcare providers, some of the HIV-positive women were unemployed due to their HIV status, and they often failed to secure money for food and transport to visit the HAART clinic. A study indicated that HIV-positive individuals sometimes forget to take their pills while working for food for a long time at different workplaces [23].

Depression appeared to be one of the major barriers to HAART medication raised by the participants in the FGDs. Research carried out among PLHIV at Gimbi General Hospital, West Ethiopia, demonstrated that depression among individuals diagnosed with HIV does not affect only their health status but also has a negative influence on HAART [24]. Another institutional-based cross-sectional study conducted among individuals diagnosed with HIV and taking HAART in Eastern Ethiopia revealed that depression was found to be a significant contributing factor to poor adherence to HAART [25].

The FGD participants showed that being busy with different activities was found to be one of the many challenges which affect the proper taking of medication among HIV-positive women. This finding was supported by a study conducted in Nigeria [26].Moreover, a systematic review conducted in 2019 showed that being busy with different activities is the main obstacle towards the effect of HIV-positive women’s adherence to HAART [27]. Forgetting the HAART doses was among the issues reported by most healthcare providers as a challenge for optimal HAART adherence. A study conducted in Iran confirmed that from a total of 122 HIV-positive individuals who participated in the study, 26.7% missed their daily HAART doses due to forgetfulness [28]. In a study conducted in Northern Ethiopia, forgetting and being away from home were the most common justifications identified for poor adherence among HIV-positive individuals receiving HAART [29]. Most healthcare providers revealed that some patients were advised to stop taking HAART after joining some religion because the pastor claimed they would pray for patients to be cured of the disease. Some HIV-positive women stop their medication entirely due to the medically wrong information from religious groups. A study conducted in Ethiopia found that seeking spiritual treatment or holy water was a contributing reason for non-adherence to HAART among HIV-positive individuals [30]. The current study also explored the side effects of HAART medication as common challenges for HIV-positive women taking HAART. A cross-sectional study conducted in North-Eastern Ethiopia shared the view that ARV side effects create a barrier for HIV-positive women to take their HAART medication regimen as prescribed by healthcare providers [31]. In another similar study carried out in South-Eastern Nigeria, side effects were found to be one of the major barriers identified to women’s HAART medication adherence. Those women who had developed side effects while on treatment were poorly adhering to their HAART medication [32]. Participants also reported that though the HIV treatment medication burden is not such a major problem in recent times, some HIV-positive women still complain about the pill burden regarding their medication. The complaint is not only associated with ARVs but also with some other additional drugs taken in parallel. If individuals diagnosed with HIV have some inconvenience with their HAART medication regimen, the likelihood of taking their medication at the right time and frequency will be reduced, which results in poor adherence to HAART medication [33]. Moreover, a study conducted in South Korea pointed out that HIV-positive individuals who are taking a low pill burden, namely, a single tablet ARV, sustain a higher level of HAART medication adherence and viral load suppression [34]. Moreover, dosage frequency was explored as a barrier to HAART adherence in this study. The healthcare participants indicated that HIV-positive individuals’ adherence to HAART can be affected by multiple frequency dosing in different ways. Challenges to integrating it into a daily routine, forgetting to take daily medication multiple times a day, and treatment fatigue are some of the potential problems that can occur by increased dosing frequency. Some kinds of HAART medication can have adverse effects, and this situation may be more pronounced during the increased frequency of medication taking time [35]. A complex medication regimen that contains multiple dosing frequencies in a day has an impact on adherence to HAART among HIV-positive individuals [36]. On the other hand, long waiting times and unavailability of services at weekends are the identified barriers by the healthcare participants in the FGDs. Clinic long waiting time is one of the conditions that potentially lead to missing or delayed clinic appointments of HIV-positive people, adversely affecting adherence to HAART. Long waiting times are challenging to people with other commitments like different family and social responsibilities, as they may be unable to allocate sufficient time to wait at the HAART clinic. HIV-positive individuals who are more satisfied with the care they receive are more likely to take their medications as prescribed, attend their scheduled appointments, have more trust in the healthcare staff, and possess better health outcomes [37]. A similar qualitative study done in Mpumalanga Province, South Africa, also reported that long waiting times of HIV-positive individuals taking HAART were a common reason for patients not achieving good adherence [38]. According to the participant healthcare providers, some HIV-positive women could not come to the clinic at the appointed time to collect their HAART medication as they were busy with their different responsibilities and commitments during working days (Monday through Friday). A qualitative study done in Ethiopia pointed out that some HIV-positive individuals who were involved in the study reported that they wanted the HAART service provision time to be on weekdays [39]. Participants described facilitators of HAART adherence as perceived benefits of HAART, family responsibility, reminders, family support, dosage formulation, experiencing improved health on HAART, relationship with healthcare providers, adherence counselling and education, and adherence supporting peer groups.

HIV-positive individuals may be motivated to take medication willingly if they observe an improvement in their health status as a result of taking the prescribed HAART medication [40]. The positive association of perceived benefits of HAART to HAART adherence has also been shown by another study [41]. On the other hand, a qualitative study conducted in Zimbabwe indicated that HAART helped HIV-positive women carry out their responsibilities as mothers in caring for their children and family members [42]. In this study, participants underlined how useful these medication reminders were for ensuring HIV-positive women’s regular and timely HAART medication intake. The importance of reminder devices in medication-taking practices has been revealed in different studies [43,44].Another important issue pointed out by participants as a facilitator of HAART adherence among HIV-Positive women was family support. Researchers have found a positive association between family support and adherence to HAART medication [45]. A study done among HIV-positive individuals revealed that participants with single tablet regimens (STR) had reported better adherence to HAART than those with multi tablet regimen (MTR) [46]. Studies indicated that HIV-positive individuals whose health condition is improved due to HAART become motivated to take their medication correctly and maintain adherence to HAART [47]. HIV-positive individuals who experience a good relationship with their healthcare providers may have a better chance of good clinic attendance and HAART medication adherence in the prescribed way [48]. In this study, it was reported that adherence counseling and education provided by the HAART clinic encouraged participants to follow their HAART medication regimen [49].

### Limitations

The study was restricted to and conducted in only three urban hospitals in one region of Ethiopia. Therefore, the findings cannot be generalized to other regions or the whole country. Another limitation of the study was the difficulty in avoiding the possibility of social desirability bias. This was also the case during the face-to-face in-depth interviews, particularly among HIV-positive women who came to HAART clinics to collect their HAART medication, which might increase the chance of interviewees answering questions with more desirable responses instead of actual answers. Despite these limitations, we used face-to-face in-depth interviews with participants who provided a rich and personal understanding of their experiences, allowing for a deeper exploration of the factors influencing adherence to HAART medication.

## Conclusions

Adherence to HAART medication is a major challenge among HIV-positive women in Southern Ethiopia. The perception of HCPs rgarding adherence among HIV-positive women need to be explored, and an understanding of the barriers and facilitators of adherence to HAART can be reached. The following were identified as barriers to HAART adherence: stigma and discrimination, gender role, lack of money for food and transport, depression, busy schedule, forgetting doses, religion, drug side-effects, pills burden and size, dosage frequency, long waiting time, and unavailability of services at weekends. However, perceived benefits of HAART, family responsibility, reminders, family support, dosage formulation, experiencing improved health on HAART, relationship with healthcare providers, adherence counselling and education, and adherence supporting peer groups. Therefore, understanding the factors that affect adherence can help in the development of tailored interventions to increase adherence and improve the health outcomes of HIV-positive women.

## Supporting information

S1 Checklist Consolidated criteria for reporting qualitative research (COREQ). A 32-item checklist for interviews and focus groups. International journal for quality in health care.
(PDF)

S1 Data Focus group discussion guide and transcription. (PDF)
