# Supplementary material for: Healthcare providers’ perspectives of barriers and facilitators to highly active antiretroviral therapy adherence among HIV-positive women in Southern Ethiopia: A qualitative study
Source: PLoS One. 2025 Sep 19;20(9):e0312980. doi: 10.1371/journal.pone.0312980 (PMC12448366; doi:10.1371/journal.pone.0312980)
Supplement: S1 Data — (PDF) [file pone.0312980.s002.pdf]

1

Verbatim transcribed-Focus Group Discussion-1

Participant: Healthcare providers (n= 8)

Abbreviations list: M: Moderator

P: Participant (P1= participant one)

Transcription results:

M: **What are the perceptions of your experiences about HAART adherence of women who are HIV positive?**

P3: Okay, as we all know when we think about HIV treatment in general, it is not as simple as we think. Because the treatment by itself needs special attention and cooperation not only from the patient but also it expects from other people including healthcare providers, family members, and others. When we come to women's HIV treatment adherence, it is seen as somehow different from other HIV-positive individuals. Women especially in our country are mostly economically dependent and are not well educated as compared to their counterpart male partners. Due to that, they are not as such focusing on their health-related issues rather they are more engaging in home activities, especially child, husband and other family members' care. HIV-positive women come to our clinic for HIV follow-up care and start to take their medication but the problem is not the initiation of the drugs. The problem with regard to their HAART adherence will come then after. Actually, there are many HIV-positive women taking their medication properly but there are also some women who are not taking their medication at the right time and the right dose. I know who are single parents i.e., single moms of children because their partners have died, they have been taking their medication daily at the right time to make themselves healthy, to work hard just to get money for their children's growth.

M: You mentioned that there are women who are not taking their medication properly and there are also not taking their medication properly. **What factors as health care providers perceive as reasons for women not taking their HAART properly? What factors do you perceive as facilitators for their medication taking?**

P7: As it is mentioned now, the adherence of women is depending upon their individual condition meaning, their economic, education and health status. Many of the HIV-positive women patients on HAART are still taking their medication according to the prescription which means during the right time and at the right dose. Some of them are not taking their ARV medication in the right manner. These HIV-positive women have different reasons for not taking their medication properly. Some of them tell you that they don't like to take their medication due to side effects, and others also tell you that they are not happy with their medication because their treatment is lifelong. On the other hand, they also tell you that they have no support, i.e., be it psychological, economic or other material. They usually tell you about the side effect they experienced from the medication they are taking and they are strictly relating the side effect to the damage to their internal organs like kidneys, liver and stomach. , therefore, they decide not to take their medication daily. To tell you frankly, some HIV-positive women stop their medication completely and they come to the clinic. When you assess their adherence you would find some underlying problem for not taking their medication. Some women explain that the side effects which occur from the ARV drugs are serious due to this they tell you that they don't like to continue taking HAART.

M: Okey, you discussed that there are two types of HIV-positive women in the follow-up of HIV treatment. How would you describe the adherence of HIV-positive women taking HAART?

P6: As it is already said, some of them are taking their medication properly and others are not taking their medication properly due to the mentioned reasons. Among the many reasons they tell you about not taking their drug are the side effect, fear of disclosure of HIV status, absence of family or husband support in taking of the ARVs, and the high number of pills are the reasons patients raised most while they come to our HAART clinic. But when we provide them with the information about their medication and adherence which need to be known by our patients, we give special attention especially to women due to their very nature of them. Most of the time those females in our nation are not as such given support from the entire community as like that of males. There are different cultural beliefs that give a special value to males than females. This situation hinders females from getting better education and better economic activities. When we see this situation with regard to the treatment of HIV, though many women are infected with HIV in our country, those who access the treatment are relatively fewer. When we observe that HIV-positive women who follow their HIV treatment in healthcare facilities are more or less not adhering well to their HAART due to different reasons like what I mentioned earlier.

P2: We healthcare providers, especially those working in the HAART clinic always think about our patients' health; this is our responsibility i.e., professional responsibility you know? Therefore, we first counsel our patients on how to start the medication and how to properly take their medication. This counselling also includes how they adhere to their medication and if not adhere to their medication what will be the conseauence and what expects from them are discussed. Then after they are also having a chance to participate in the decision which will be made on the initiation of the ARVs. After they have already come up with the consensence we advised them to start their medication. The major issue here is the explanation of HAART benefits and the expected side effects from the ARV drugs are given special attention by the healthcare workers and thus these all issues are briefly discussed with the patient. Finally, they start taking the medications prescribed. Then after they follow their schedule to visit the HAART clinic just to check for any health-related issues, and refill their medication.

P10: HAART is the backbone of HIV treatment as this improves the health of HIV-positive people as there is no other option other than this treatment. Due to this currently, many HIV-positive individuals are following their HIV treatment in different healthcare facilities including public hospitals and health centres. As one objective of the HAART is just to reduce the viral load of the patient and then improve the health condition of the HIV positive individuals including HIV-positive women. Therefore, HAART is highly important for HIV patients throughout the world including our country. The Federal Ministry of Health has provided us with different knowledge and skill training related to HIV treatment.

M: You said that HIV-positive women's better health condition was due to the support of the ARV medication? Would you please explain this more?

P1: Except for some HIV-positive women, the majority of them know their health condition has got improvement due to the ARVs they have been taking. We as healthcare workers know what is the benefit of HAART. But what I want to say now is from the perception of our patients about HAART. Therefore, when those female HIV-positive patients tell us the benefit they have gotten from the medication which they take daily. We also assess the patients clinically, immunologically and physically just to know their health condition while taking the ARV medication. So what we observe is there are women who take their medication according to the prescription on a daily basis and there are also some other HIV-positive women who don’t take their medication properly. The health condition we see on them is also different meaning those who strictly take their

ARVs are in a better health condition and on the contrary, those who don’t take their medication properly have not good health condition.

M: What and how do they tell you about their HAART and other related issues like adherence and what is your perception on their adherence to HAART?

P7: In the first place, being a woman by itself has its own drawback when we think of HIV and its treatment. Gender has a vital role in our community and it explains the negative impact on women's HIV treatment. Due to our community's cultural norms, women are placed behind their counterparts. Women have a strong responsibility to their entire family and community. They are expected to provide care for their home, children, husband, family members especially for aged persons, and others accordingly. For example, …in our community men have the right to go here and there without any explanation about where they are going; therefore, they can go to the hospital to get a diagnosis and treatment easily, but women are not in a position to go anywhere without detail explanation and notification about where they want to go to their partners so that they couldn’t go HAART clinic easily to collect their medication timely. Therefore, such a kind of gender-related issue can create influence on women's HAART adherence.

P3: As a healthcare provider, I have also understood that gender norms in our culture have an influence on women's health-seeking behaviour. Look! There are some women with children who have difficulties in their day-to-day lives and medication-taking efforts. I know an HIV-positive woman in my encounter who has two children with her. She is a single mother, she always takes her children to school early in the morning and brings them back to their home late afternoon. She always provides care for them and does everything around the home. She sometimes couldn’t take her pills. It is very challenging for HIV-positive women to take their HAART regularly as it has been prescribed because of their multiple roles in their family and community.

P4: Yes, gender-based issues are sensitive for women who are HIV positive. Actually, issues like gender-based violence occurring by their partners, and gender-based roles and responsibilities of females are very common issues that impede adherence to HAART of the HIV positive women. Because of the community’s cultural norm, it is already accepted that every woman and their partner should be obeyed by this norm. therefore, women in our community are expected to spend time more in their homes and villages by taking care of their children, partners, and family members and by doing activities around their homes and village. Due to that those HIV-positive women may not have a chance to visit even the nearby healthcare facility for their illnesses and

treatment. Therefore, this gender-based cultural norm has an implication for HAART adherence of those HIV-positive women taking HAART.

P10: Yes this is a common challenge that we have seen daily in our work. Many HIV-positive female patients usually forget their dose while they are engaged in their home activities. Especially those housewives have many activities to do as they are the only ones responsible for their family, especially their children, husband and other family members. These HIV-positive women are also busy with their social activities in their village, for example, they may participate in Idir gatherings during the death of community members. Therefore, they are sometimes busy and vulnerable to missing their dose as we have seen in their life experience.

P1: There are HIV-positive women who come to the HAART clinic for their medication refilling tell us about their adherence clearly. Some women have different opinions about the medication they are taking. For example, some of them explain to us their concern about lifelong treatment. They don't like to take their medication because of the nature of the treatment as this is a lifelong treatment. Therefore, they complain about the huge number of pills they are taking daily and this makes them fatigued thus some of them decide to take their medication by skipping their dose you know. The other issue these patients often raise is the side effect of the medication they are exposed to. In this regard, many patients tell us that they observe different side effects while they are taking the medication. They tell us about the problem they encountered due to taking their medication. Usually, these patients complain about nausea, vomiting, headache and so on. Therefore, the side effects of their medication and the number of pills they have been taking are the major raised issues by our HIV-positive women. These problems are also the reason for not taking their medications properly.

P3: For me, as a physician, I understand that there are diversified reasons for HIV-positive women on HAART to be adherent and non-adherent to their medication. In this regard, the problems they mentioned that impede patients from taking their medication correctly, for example, fear of disclosure of HIV status due to stigma, side effects and economic problems are the major ones. On the other hand for those who are taking their medication properly, things like family support, reminders, and perceived benefits of HAART are mostly facilitators for their adherence to HAART. As a physician, I always assess my patients for their understanding of their medication and adherence and I get their concerns and any issues they have about their treatment, facilitators and challenges for their

adherence to HAART. The good thing here is there are also HIV-positive women who are taking their medication correctly and thus, they are in good health condition.

P9: As mentioned there are HIV-positive women who have been taking their medication at the right time due to their effort and conducive environment just to take their medication. There are reasons to be mentioned here for these patients, for example, they use a reminder to take their pills. Since not all HIV-positive women are well educated and have mobile phones, those patients who have no mobile phones and are not able to operate phones, usually use the local factories' bell rings, and mosques' Adhan sounds as reminders of the right time to swallow their pills.

M: Would you please make it clear one by one; how the mentioned reasons are facilitators and challenges for HIV positive Women to their HAART adherence?

P8: Yes as it has been said, some patients raise complaints related to side effects. They tell us that they have developed different side effects like diarrhoea, abdominal cramp, skin rash, nausea, and vomiting. Though we advised our patients not to discontinue, or skip any dose due to some temporary side effects, some of them still don't like to continue their medication after they have developed some of the mentioned side effects due to this they are unable to adhere to their HAART. In general, some HIV-positive women discontinue or skip some of their doses due to the HAART side effect. This is the common reason for patients not to be adherent to their medication. On the other side, our patients usually mention that they are stigmatized by their family, friends, employer, and other community members due to their HIV status. Because of the fear of stigma, patients don't like to take their medication in the presence of others even during the right time for their medication taking thus they would miss or skip their dose.

P10: Okay, like that of the challenges which make patients not take their medication properly, there are also some important factors that help our women patients to take their medication properly. Some of the facilitators which help patients take their ARV pills daily at the right time are family support, understanding of patients on the benefit of HAART, and even the regimen which contains single or fixed tablets. Those who take their medication properly tell you that they are so happy with the one-pill-per-day regimen because it reduces their previous pill burden. And as a healthcare provider, I understand that one pill or fixed tablet which contains two or more drugs in the form of a fixed tablet is helpful for better adherence to HAART. On the other hand, if those patients get any kind of support from their family, relatives and friends, they are comfortable taking their medication properly. Especially they might not be stigmatized and thus they can

take their medication easily in front of their family or friends at the right time even without the fear of stigma. Generally, I have understood that patients have challenges and facilitators to take their medications properly. Those patients with the above-mentioned challenges like fear of side effects, stigma, pill burden, and lack of social and family support are not taking their ARV medication daily at the right time. Some HIV-positive women disclosed their HIV status to their families and get support from them. There are women who get support from their husbands. I remember one HIV-positive adherent women who tell me that her discordant husband and their children love and support her to take her medication at the right time. When she visits our clinic she always tells me about the support she has from their family and this makes her have good adherence to her medication.

P9: In our HAART clinic, there are many patients following their HIV treatment. The majority of them are females and are of childbearing age. As we healthcare providers know especially women have many challenges in the taking of their medication as compared to their counterparts. From the many challenges they have faced, they are busy with their day-to-day activities either in the home or outside the home activities where they are employed. There are also women who don't want to disclose their HIV status to anyone even their beloved ones. Sometimes there are women patients taking HAART, who don't come to the clinic on the scheduled date rather they may come after the clinic scheduled date and tell us that they were busy with their routine work as a reason for not coming on the appointment date. They are so busy in their home activities as most of them are responsible for caring for their family including their children, husbands, and other family members. This responsibility has created a huge burden on their personal life especially related to their health. By the way, in addition to this which means their home responsibility, many women are working outside either private or government organisations, or as daily labourers in private individual homes just to get some income for themselves and their families. Through this process, they may not take their medication properly which means at the right time to take their medication. Therefore, being busy with their different activities make HIV positive women not take their medication properly similarly if they are busy with their either home or outside activities, they would not come to the HAART clinic on the scheduled date to collect their medication and this, in turn, make them not to adhere to their clinic schedule and HAART.

M: Good, you have been telling us some of the challenges of HIV-positive women in their medication-taking process, from these you mentioned, drug side effects, fear of disclosure of HIV status, lack of social support, pill burden, and being

busy with different activities and so on. Would you please tell me more in this regard?

P1: Yes, it is common that patients to forget to take their medications due to different personal, family and social reasons. The common reasons that patients mention always are; for instance, travel to somewhere away from their home, engaging in different social affairs (weddings, funerals, social gatherings and others), and personal reasons. During these circumstances, there are some patients who forget to take their pills at the right time. It is known well that our culture gives an emphasis on social affairs like attending any community gatherings which include wedding ceremonies, funeral ceremonies, religious gatherings, and any related family ceremonial issues. During all these social affairs, HIV-positive women may go from one place or town to somewheree else which is far from their home. When they travel to another place they may forget to take their medication with them. In addition to this, they may have no time or comfortable situations to take their medication at the right time on a daily basis. Therefore, they usually miss some doses or interrupt taking their medication for days or weeks even. So being busy with social affairs and away from their home due to the mentioned reasons have a negative impact on their medication-taking schedule. This is what I have observed in my encounter with some HIV-positive women. Based on this we always try to address such issues through our adherence counseling sessions. We always discuss such issues with our HIV-positive women in the manner of setting strategies on how they try to take their medication while they will be in any social engagement. What I have mentioned here are only the challenges of some HIV-positive women taking HAART. There are also positive aspects that help HIV-positive women to take their medication properly, anyways I will talk about this issue later. Patients tell us that they have changed their negative thought about HAART after they have been engaged in adherence and counseling provided by healthcare providers at the HAART clinic.

P5: Yes, these people which means HIV positive women taking HAART, as human beings, especially as a female and home caring person, most of the time they are busy with their home activities because, in our country and culture, they are the front and the first person to shoulder the caring of family. They are mothers, and expected to take care of their children, as a wife they are also expected to take care of their husbands. Therefore, they are so busy with their different home activities. In addition to this, they [HIV-positive women taking HAART] are highly expected to engage in any social affairs, like wedding ceremonies, and funeral ceremonies as my colleagues said. The reason that I have raised this specific issue in this discussion is I remember one recent HIV-positive woman history I heard during her unscheduled clinic attendance for her acute illness. I understood that she was not at her home for some days due to social reasons. She told me that she went to another town which is almost 275 km away from her

residence to attend a funeral ceremony as her best friend's father had died. As she was in a hurry, she forgot to take her ARV medication with her and she stayed there for five days without taking her pills.

P4: Yes, being busy and forgetting doses are the common problems which have been mentioned by some HIV-positive women. These HIV-positive women tell us different issues as reasons for forgetting their doses during their routine life. From these, forgetting their dose due to a busy schedule is the major one. Most women are still responsible for their families. They prepare food for their children, husbands and other family members. During their engagement in-home activities they may forget to take their medication and thus the right time for taking their medication would pass as they are busy with home activities. But there are also some other reasons for women patients not to take their medication properly. Some HIV-positive women don't want to take their medication with their religious-related issues. This is also another common factor mentioned by HIV-positive women. There are HIV-positive women who discontinue their medication while they are under their religious rituals. They usually tell us that if they are in a religious formality, they are not required to take their medication because of the doctrine which forbids them not to take any scientific or local healers made medication. In addition to that, fasting is also related to the above doctrine. If individuals are on fasting during the religious fasting seasons, they are expected not to take any medications other than some kind of food and water. During their fasting time, they are highly expected to pray rather than take any kind of medication. Therefore, if an HIV-positive woman taking HAART, is on such religious fasting, she doesn't take her ARVs because of the doctrine they must obey. Due to this, some HIV-positive women skip their doses or discontinue their medication for weeks or months.

P9: Especially, some HIV-positive women tell us some issues as their reason for not taking their medication as per the recommendation. From these issues or reasons, religious rituals like fasting and holy water are the major ones. Those Christian orthodox women with HIV positive taking HAART, usually tell us they would not take their medication during their fasting and if they start to a religious treatment by holy water.

I remember one HIV-positive woman who discontinued her medication for 6 months due to the holy water treatment. She came to our clinic just by tracing her through the adherence supporters of the hospital. She told me that she doesn't like to restart her medication as she had already been cured of HIV by holy water.

P5: This is the common issue of most patients including female patients, I also find some HIV women-positive patients. Women are not always busy in their home activities or other business activities they are also highly involved in religious rituals. Some women patients do not believe in HAART. They believe that they will be cured or have better health conditions by their spiritual belief than by their medication. Due to that even though they are taking their medication, they think that their religious ritual has more benefit than the medication. Therefore, from the challenges which impede HIV-positive women from taking their medication as per the recommended dose and time, religious beliefs are the common ones. Before one year, one HIV-positive woman who follows her HIV treatment in our clinic told me that after a week's prayer for her, the pastor told her that she had been cured and thus he ordered her to throw her medication forever.

P10: Economic problem is also another reason for women patients that creates influence their medication-taking process. Some women are not in a position to support themselves with money. Women patients who have no economic support are highly complaining about lack of nutrition, house rent and transportation. Some women have a transportation problem and even to come to the clinic on their scheduled date due to the lack of money thus, they sometimes aren't able to collect their medication from the clinic at the right time; finally, they have no medication to take at the right time. If they don't have food to eat, they don't like to take their medication on an empty stomach. Some of them also tell us that they couldn't take their medication properly because of the lack of nutrition due to a shortage of money. It is obvious that most of our country's women are not well educated as in other developed countries women and due to this, they have no as such better work for generating money. Due to their less level of education, some HIV-positive women are working as labourers and getting some money which is probably not enough to cover their family expenses. This is also what we have seen in some of our HIV-positive women taking HAART. Therefore, if they don't have money for transportation to and from the HAART clinic they wouldn't attend and collect their medication from the clinic on time and if they don't collect their medication during the scheduled date they will not have medication at hand to take at the right time.

P6: When we communicate and discuss different treatment issues, especially with non-adherent HIV-positive women, they usually tell you that one of the reasons for not taking their medication at the right time is the lack of money for their day-to-day expenses, house rent, nutrition, transportation to come to the clinic and so on. Previously there were NGOs working on the support of HIV-positive people in money and other items but nowadays these NGOs are not existing as previously. Due to that those HIV-positive women who were supported by these NGOs

currently have an economic problem as the support has stopped. As previously discussed some HIV-positive women are working as daily labourers in different working conditions. The problem here is the money they get from such working situations is not enough to cover their needs. Due to that, they have no money for food. Unless they get enough food for their life they discontinued their medication as they have an understanding that taking HAART without nutrition may cause severe body damage. Moreover, as it is known, ARV medications are given to patients free of charge, but there are other related costs which need to be covered by patients. Patients need to pay for many other laboratory tests and related medical services. Some patients become angry due to the payment when requested to pay for the service and due to this, they might return to their homes without collecting their ARVs.

P2: With regard to the economic problem, some patients tell us that they have no money as most of them are not employed because they are not educated well. If they get a job they will work with a low wage rate and work for long hours. Due to that, they can't afford the expenses for food and transportation to come to the HAART clinic.

M: You mentioned that economic problem is one challenge for non-adherent HIV-positive women. How would you identify non-adherent women? Can you explain the concept of adherent and non-adherent patientss?

P10: Okay, through our routine work, as a healthcare provider, we always assess our patients' adherence level through standard procedures or mechanisms. From the mechanism that we assess our patients' adherence level, WHO and national HIV treatment guidelines are the major ones. These HIV patients' treatment guidelines are almost similar except the national guideline has some country-oriented approach to the implementation of the approaches. Otherwise, all the major treatment approaches are similar. As a standard national HIV treatment guidelines are developed by adopting and modifying the WHO HIV treatment guidelines. Therefore, we assess patients through clinical, immunological and by patients' medication taking history. Based on these, each patient has their medical and treatment history which has been captured always during the time of HAART clinic visits. We have strictly followed our patients' medication practice by asking them how they have been taking their pills. This is a routine practice of our clinic.

P3: Yes, though it is subjective, the simplest way of assessing our patients' (including HIV-positive women) medication-taking practice is by asking them how they are taking their medication like at what time, how many doses and how frequently they have been taking. Then patients might tell you about their experiences one

by one. Actually, some patients may still have some information about their medication-taking practice but the way we approach them should be more polite, supportive and ethical; then they could be free to tell us their practice about taking of ARVs.

M: You are discussing some issues which affect the medication-taking practice of HIV-positive women; would you add more please if there are others to be mentioned here?

P5: Another major influencing factor for HIV-positive women not to take their medication properly is the drug side effect. In the majority of cases, they [HIV-positive women taking HAART] complain about the side effect caused by the ARV medication they have been taking. To tell you the truth, this issue which means the side effect occurs by ARVs is the leading cause of HAART discontinuation or skipping of doses which has been observed in patients taking HAART. In my day-to-day practice, I have seen many HIV-positive women raise the issue of side effects which occurred by ARVs. These women who are taking HAART usually tell you that they have experienced different side effects including headache, nausea, abdominal discomfort, dizziness, nightmare, etc., Some women come to our clinic after they have discontinued their medication due to the side effects they appreciate. Some of them even disappear from their clinic follow-up care because they have no interest to continue their medication as they have developed a fear of side effects secondary to the ARVs.

P8: It is a common issue of our patients, there are also some women patients who complain of severe side effects of ARVs like the disproportion of body fat which they explain as their abdomen becomes big, their face and buttock areas become thin and thus they have a great concern about their HAART. Actually, as healthcare providers, we advise them not to discontinue as most of the side effects are temporary and will disappear after a few weeks. On top of that during the initiation of their medication, we provide them such detailed information and also we provide continuous adherence conseling for them. But some women patients don't accept such common and temporary side effects and for some patients, it is the reason for non-adherence to their medication.

P7: These days as the preparation of ARVs is in the form of a fixed tablet, the challenge of each individual drug side effect becomes reduced. But as mentioned earlier the issue of side effects is highly influential in the process of proper medication-taking practice. Some patients tell us the fact about their ART experiences without fear. For instance, one of our female patients told me that she was thin just like ... after she had started her ARV medications her body became fat. But the worst thing that came along with her fatness was, she turned to bedridden due to the side effects of her medications [ARVs] which include vomiting, nightmare, dizziness, and so on. She just decided to cut them off taking

and went to one known church for praying. The pastor whom she knew before, prayed for her for three weeks, and then her health became better ... she thanked that pastor, next to God. She also told me that she had taken her medications every other day with strong praying and that made her fine.

P9: With regard to the side effect, as mentioned in detail, different HIV-positive individuals including women have different opinions. Some women take this side effect as a life and death issue and have taken this issue as a serious challenge which prevents them from taking their medication properly. Therefore, there are some patients who discontinue their medication after they have started. This is one of the reasons for HIV-positive women not to take their medication at the right time on a daily basis. Because women are highly worried about their shape than that of males the probability of discontinuing their medication due to this reason is high. We always have to try to address such kind of false information during the routine adherence conseling sessions. In addition to that, there is also an **adherence supports peer group** here in our hospital and thus the member of this group has always been communicating with non-adherent patients just to share their [**adherence supports peer group**] experience with regard to HAART. But still, there are patients who don't like to give attention to the healthcare provider's advice, and information about the overall treatment process.

P4: I have also recognized that some female patients complain about ARV drug side effects. They explain the type of side effects they have seen in other patients as like as they have already been exposed to such side effects while they are taking their medication. Due to this misunderstanding of exaggerated side effects, some HIV-positive women patients, discontinue their medication or take it with some days or weeks interruption without telling or discussing the issue with us. This kind of self-decision of medication interruption or discontinuation has been seen in some HIV-positive women as well as men. A female patient told me that "My body shape became changed after I had been taking my ARVs for one year… Doctor, please change these medications otherwise I have already decided not to take them unless they could be changed. My husband always tells me that my body shape has changed and thus he would not continue [as a husband] with me. I don't know why this happened only to females. My husband also taking his medications but there is no anybody change due to these medications". On the other hand, some other additional challenges influence proper medication-taking behaviour. These include pill burden, patients sometimes have a misunderstanding of HIV and pill size. These all the stated factors most of the time are mentioned by our women HIV-positive patients and also by male patients. But what I understand from my work experiences the issues related to

poor adherence are usually mentioned by women as they are more responsive to their children, husband and other family members.

M: You are telling us about the challenges which affect the correct taking of medication of HIV-positive women on HAART. From these you mentioned pill burden, pill size, and misunderstanding of HIV; can you discuss more how these issues affect HIV-positive women medication-taking practice?

P2: As it has been mentioned some female patients who are taking HAART have a misunderstanding of HIV. As we all know it has been now more than three decades since HIV become the problem of human beings. In addition to that, there is also nothing that can cure HIV till now. When we think about HAART, it is a combination of different ARVs used for the treatment of HIV by reducing and averting the multiplication of the virus in the human body but this treatment doesn't have a curing effect. Therefore, if one HIV-positive individual start taking HAART, she should take the medication lifelong. Though that is the reality, there are some HIV-positive women who have a misunderstanding that HIV may affect individuals by chance and if someone is infected with HIV and started taking HAART, it is possible that this medication can be taken with interruption or skipping some doses. Some female patients still believe that HIV can be cured due to religion or some other unknown reason and miracle. Due to that even though these patients have started to take HAART, they will stop their medication at some point in their life. The reason for their medication interruption or discontinuation is because they have an understanding that HIV can be cured due to different reasons like religion or any miracle. In this regard, we had different and many occasions that we discussed such issues with the patients who already had such understandings. I remember a woman who came to the HAART clinic with severe weight loss and cough after she has lost and discontinued her ARVs for more than a year. She believed that she was free from HIV because she was informed by a pastor of one church as she had already been cured of the virus [HIV] because of the prayer she did.

P10: Pills burden and size are some of the challenges to hinder the proper taking of medication which mention always by our patients. Yes, some patients complain about the burden they have due to the pills taken daily for a long time. Due to that, they tell us that they have a load from a daily long time taking pills and thus, they sometimes miss their doses.

P1: Yes such issues are very common among our female patients especially who have a history of poor adherence to their medication. As previously mentioned that there are some patients who believe that HIV is a kind of simple virus or infection which could be cured through their religious belief or spiritual belief. Those patients including some HIV-positive women are taking their medication appropriately and they have no trust in HAART because they considered it as a temporary solution with many challenges like side effects, at the end of the day their adherence to their medication would be very poor. Let me tell you about a recent case related to this issue. Women with her little sister came to our HAART clinic almost two months after her appointment date. I asked her the reason for her missed clinic appointment date. She started to talk with high confidence that she became cured and due to that, she doesn't want to come to the clinic. The reason she mentioned her cure from HIV was the prayer which was done by her religious colleagues and pastors in their known church. Therefore, this is an indication of the poor level of understanding of HIV infection as they think that it can be cured which is contradictory to the existing facts about HIV and treatment.

M: How would you explain the HIV-positive women's adherence to their treatment?

P3: As we have discussed many determinants or factors which could affect the adherence of women who are taking medication. These challenges of factors directly or indirectly create pressure on these patients not to take their medication accordingly which means properly. It is known that there are more female patients who are following their treatment for HIV in our clinics, is that not? Yes, it is true because the burden of HIV within African countries including our country, Ethiopia, has affected females more than their counterparts, males. This also happens which means the number of HIV-positive female patients is more than that of HIV-positive males. Due to that, we have more HIV-positive female cases in our clinic and from the day-to-day adherence assessment we conduct we understand that many HIV-positive women taking HAART have poor adherence, you know?

P6: In our HAART clinic also the number of HIV-positive women is more as compared to the number of HIV-positive men. As it has been said, this is almost similar in most of the healthcare facilities in our country. There are also many HIV-positive female patients who have poor adherence to their medication as we observed from our assessment being done in the clinic. Therefore, though the majority of HIV-positive women who are taking the ARVs have good medication-taking practices, it is also possible to say that not a few HIV-positive women have substandard or poor adherence to their medication.

M: could you tell me more about the adherence level of the female HIV-positive individuals you observe?

P10: For me, it is impossible to put in numbers how many of the female patients have poor adherence or have good adherence. but the one thing that can be mentioned here is, there are many HIV-positive women who don't take their medication appropriately. This poor adherence is related to the reasons we mentioned before, like side effects, pill burden, fear of HIV status disclosure to avoid social stigma and discrimination, economic problems, lack of sufficient knowledge on HIV and so on major factors. But the majority of the HIV-positive women have good adherence still there are many numbers of HIV-positive women who still have poor adherence and it needs further strategies which can be developed based on the findings of such a kind of this research.

M: You have been telling us the barriers/challenges to women's adherence to HAART. What do you think are the facilitators of HIV-positive women's adherence to HAART?

P9: Fine. As my colleague said there are also many HIV-positive women who have a good adherence to their medication. This is what we want to be on our patients either males or females. But as you know females are more vulnerable to HIV infection as well as its effect on them. This is due to natural/biological, treatment-related, social, and economic factors. Therefore, we better focus on the female or women HIV positive individuals who are taking HAART. on top of that the very important thing here is that the reason for our focus group discussion is the issue with regard to HAART adherence of HIV-positive women. So having considered this, I will reflect on my idea related to the issue. For those HIV-positive women who are taking their medication at the right time and the right dose, there are some facilitators to this medication-taking practice to be mentioned here based on my experience. Those women who have good family support could be taken their medication properly. This family support may be explained in terms of psychological support given to the women while taking their medication, and economic support from their family members like to cover different expenses including nutrition, house rent, transportation and medical expense other than HAART. I know [in HAART clinic] many HIV-positive women taking their medication who get support from their family without the fear of stigma and discrimination. AS these HIV-positive women have already disclosed their HIV status to their family, they can take their medication in front of their family members and don't have any fear of stigma by their family thus they can take their medication at the right time without interruption or missing doses.

P8: Yes, support from family, relatives, and friends is highly important for those HIV-positive individuals. If patients don't have support which is any kind, their thinking toward them has become distorted. They considered themselves as being unwanted and avoided persons. Their mental is not stable and couldn't perform any routine tasks. They might be restless and stressed due to a lack of support. In this regard, what I observe from these HIV-positive women who have any kind of support from their husbands or other family members including their children, they are stable and taking their medication properly as intended. The support they get from their family covers psychological support, reminding them to take their medication at the right time, and providing money for transportation to come to the clinic during their appointment.

P9: Family support is highly important to adherence to HAART because one of the problems which we always observe in our patients is a lack of family support. Those patients who have support from their family have better HAART adherence than those patients with no family support. The support may be emotional, economical and other kinds, therefore; it gives patients hope, and also help them establish good relationships with their family. Finally, this creates a relaxed condition for their medication-taking practices.

P4: These days, as we all know the pill type for HIV treatment has been changed, meaning most of the previous ARV drugs are given individually as separate tablets but currently there is a new drug formulation made by fixing two or three drugs into one tablet form. This drug formulation or regimen is reducing the complaints related to pill burden which was raised by patients who are taking HAART. Previously, one patient need to take at least two to three tablets two times a day and if any additional medication like Bactrim, anti-TB, and other drugs was ordered for other illnesses the number of tablets taken in a single day by patients would be higher and this made patients not comfortable with the medication they had taken. Currently, most of the HIV-positive women who are taking HAART are taking one or two single tablets per day for HIV treatment and this is convenient for them due to this they tell us one of the reasons to take their medication properly is because of a simple drug regimen which is easy to take it daily at the right time. I remember many patients discontinued their medication as they had an excessive pill to swallow daily. They complained that they had nausea, heartburn, and abdominal distention, for example. This is currently not a major issue for patients to stop, discontinue or skip doses of the medication. What I have understood in general, a single or fixed form of ARV is very

convenient for HIV-positive women to take it easily and in turn, it helps patients to adhere to their medication.

P2: We observe our patients, especially those HIV-positive women who are taking HAART, practice with regard to how and under what condition they are taking their medication. Through our day-to-day follow-up of patients' adherence, we understand that those HIV-positive women are adherent to their medication due to the simple regimen that they have taken. Not only a single ARV medication would be taken by HIV-positive women but also the frequency of drug taking in a day is reduced due to the regimen. For example, if we remember the previous experience of patients, they were expected to take their medication two times a day but most of the single pills are taken once a day. Therefore, fixed pills and the less frequency of dose taking the time make HIV-positive females take their medication properly and adhere to HAART.

P4: Yes, the secret of most female patients becoming adherent to their medication is multifaceted. These days many HIV-positive women have noticed the benefit they get from HAART. We know there are HIV-positive women who have not yet understood the benefit of HAART. To the contrary many HIV-positive women. tell you that they have been improved especially in terms of their health. Some patients also express their feeling toward the HAART as a lifesaver and the reason they have taken their medication is just to keep their health better. They also informed us that if they hadn’t taken their medication properly as recommended, they wouldn’t have been healthy and hard work like this. Therefore, many HIV-positive women are taking their medication properly as they understand the benefit they have been getting from HAART and finally, they have good adherence to the HAART.

P9: As has been explained by my colleagues, I have many HIV-positive females who have been taking their medication with great caution meaning with the correct dose at the right time. These women always come to our HAART clinic with their appointment date so that they can collect their medication before runs out of the previous medication at hand. They are very comfortable with the ARV medication they have been taking because of the knowledge they have about the importance of HAART. The good thing here is, the majority of HIV-positive women have a better understanding of the benefits of HAART and thus they tell us how their health get improved from their low immunity and illness which occurred before they started taking their HAART. Some of them tell you that they were bedridden, weak and couldn’t perform their routine activities. Currently, after they have

started taking their medication, their health condition has become improved, they are strong and can do their daily activities because of the HAART.

P5: Some HIV-positive females have some discomfort with HAART, for example, side effects, the lifelong treatment of HAART, and the likes but they still take their medication properly and they are good adherent to their medication. Do you know why this happened to them? Yes, they have now more understanding that HAART improves their health condition and is the only option to control the multiplication of the virus in their body.

P3: As previously mentioned by my colleagues, there are many women who are taking their HAART and tell us many times about the benefit of HAART they get. Some of them were very sick and even bedridden for some time due to the illnesses as their immunity had declined severely because of HIV. Illnesses like lung TB, PCP which is the worst type of pneumonia, and other mental-related conditions. As we observe and these women tell us, their health conditions increasingly become better. This is due to the action of HAART on their body. Therefore, many women tell us that they are comfortable with the medication i.e., HAART they are taking. The routine adherence counselling services provides to patients in the HAART clinic, could create a better understanding of the benefit of HAART. Not only the counselling session but also those the hospital **adherence supports peer group** groups are highly important in creating and increasing women's knowledge about HAART. Women who are taking HAART also mention always the helpful conseling and experience sharing they have gotten from the **adherence support peer group**. They are so important for our patients because these peer-supporting group members are all females and have good adherence to their HAART. They share their life experiences with patients. Always, they also create a mini-discussion forum for patients who are newly initiated HAART to discuss any individual and treatment-related issues and thus this helps these HIV-positive women to have a better adherence to their HAART.

P1: Yes those adherence peer support group is available in our hospital and working hard as a healthcare provider, especially in supporting those women to have good adherence to their HAART. Many HIV-positive women who have good adherence to their medication tell us the benefit they have gotten from this adherence-supporting peer group. They are so important for HIV-positive women because these peer-supporting group members are all females and have good adherence to their HAART. They share their life experiences with patients. Always, they also create a mini-discussion forum for patients who are newly initiated HAART to discuss any individual and treatment-related issues.

M: You have told us about what looks like the experience of HIV-positive women to HAART. From the information you gave us the adherence challenges those women faced are side effects, stigma and discrimination, pill burden, economic problem (lack of money), religious rituals, being busy and forgetting doses are some of them which prevent those women from taking their medication properly. On the other side, you have also discussed some of the adherence facilitators which help women to take their medication properly and become adherent to their HAART: family support, single pill and easy regimen, women understanding of the benefits of HAART, and **adherence supports peer group**.

Do you have any additional ideas or information to be added? Anything you want to add and make it clear?

We have finished our FGDs. I thank you very much for taking the time to participate and for the valuable discussion you made.

END OF INTERVIEW.

2

Verbatim transcribed-Focus Group Discussion-2

<u>Transcription results:</u>

M: **What are the perceptions of your experiences about HAART adherence of women who are HIV positive?**

P5: Well, as it has been explained by the researchers, this focus discussion on HAART, especially focusing on women's adherence to HAART, is very crucial because as we all healthcare providers know most of the time those female individuals who are diagnosed with HIV are more infected with and affected by HIV. We always see what the female patients' HAART adherence looks like. Therefore, we know the factors which challenge these patients in their daily HIV medication-taking practice. We observe different challenges in these female patients. Similarly, we also understand what factors facilitate the proper medication-taking practices of women. Based on that I want to say something related to my observation. HAART medication-taking practice is not an easy task for individual patients and also healthcare providers. the reason why I have been saying this is, HAART is not one drug but it is two or more drugs are given for the treatment. Due to that patients are usually not comfortable taking it as another

kind of medication. There are some patients who complain about the side effect of HAART. Some women who are taking their medication sometimes skip their doses because of the fear of the side effect of the drug they have been taking. As I have said, these patients tell you that they have experienced different adverse effects like dizziness, heartburn, abdominal discomfort and headache. Actually, before and during the initiation of HAART we informed them that there may be some kind of side effects while they are taking the ARVs. We also tell them not to interrupt or discontinue the medication due to side effects as the majority of side effects are self-limited and may not stay long. Therefore, we always tell them not to interrupt or skip their medication but still, some patients are taking this medication by skipping or interrupting some doses of the drug. A side effect of ARVs is not the only reason for women not taking their medication properly. There are other additional challenges which make these women not take the medication. Among these challenges, some of them are a long time that the medication needs to be taken, fear of disclosing HIV status to avoid stigma and discrimination, the number of pills to be taken and economic problems. Busy schedules and forgetting the doses are also the reasons for HIV-positive women not taking the medication according to the recommended doses and at the right time.

P8: The reasons which interfere with the proper taking of mediation that are mentioned by our patients are many. There are women patients who attend the clinic scheduled timely. But there are also patients who do not want to come to the clinic just to collect their medication in a timely manner. Women are highly burdened on their home activities as they are very responsible for their entire family. They are expecting to care for their children, husbands and even other family members. They are the major responsible person for the home management of their counterparts. They are also expected to support the home or the family economically. On the other hand, in our country women are not well educated as compared to men. These all circumstances make HIV-positive women taking HAART very busy. As they are busy they might not remember their medication to take timely. Similarly, if they don’t remember the right time to take their medication doses would be missed. In general, what I have understood, being busy with home or outside activities makes HIV-positive women forget their doses and finally they skip the doses. This is the common reason for not taking their medication at the right time. the other common reason that I have seen on some HIV-positive women is they don’t have an interest to take HAART as a whole because they wrongly think that HAART is not effective in women rather it makes their body shape bad. Therefore, they don’t have to accept the importance of HAART in their body. As we are always challenged by such a kind of wrong outlooks of HAART. We always provide our patients with

different important information just to have a better understanding of their medication and adherence. most HIV-positive women are on a good track to taking their medication appropriately. This is due to their level of understanding and the information and the medical advice that we provide them. Due to this most females are changed their wrong perception or attitude on the benefit of HAART as well as adherence to the medication.

P7: When we think about the adherence of patients including women, we can mention many kinds as we already know that they are interfering with the women's proper medication-taking practices. Some women are very much interested in what they are taking and they are good adherents to the HAART they have been taking. This is due to the understanding of women on the benefit of HAART. There are many women who are taking HAART and tell you that their health condition is highly improved due to the medication. because of the benefit they have got, these women take their medication at the right time and at the right dose as much as possible. Those women who are taking HAART and have family support are taking their medication according to the recommended dose and time. Therefore, women who are taking HAART are dependent on their entire condition and whether to take their HAART properly or not. These depend on their surrounding factors or situations. That is the reason some of the patients have good adherence and some of them have bad adherence to HAART.

M: Okay, you are discussing some factors which make HIV-positive women taking HAART take their medication properly. And some of the challenges hamper these women from the correct taking of HAART. Would you please tell me what conditions make women not take their medication correctly?

P9: As a healthcare providers, we understand that HIV-positive women have different challenges which prevent them from the proper taking of HAART. The first and most challenging is the ARVs side effect. this is the major reason for those women not taking their medication as intended. The majority of side effects are mostly temporary and would subside in a few weeks or months. Some HIV-positive women come to our clinic and tell us they have already discontinued their medication due to the side effect they developed due to ARVs. Some of the women even may not come after they have developed some side effects because they considered that HAART is not important and convenient to them and they go to the final decision that they must stop their medication. Even very trivial and temporary side effects are taken as a very damaging problem by some women taking HAART and this is seen as a means of drug discontinuation. On the other hand, there are also some women who simply want to take their HAART by skipping some doses in between their regular treatments. These women believe that the daily taking of HAART is not good for their bodies rather

taking HAART with skipping some pills is enough to control HIV in their body. Due to that, they have already developed the irregular medication-taking practice to the contrary of taking the medication regularly i.e., the right dose during the right time. These days many HIV-positive women taking ARVs have understood that HAART is highly important for their life. Actually, the fear of side effects is still there but as they have already understood that these side effects will disappear after some weeks or months they prefer not to miss or discontinue their medication rather they continued taking them because of the benefit they get from HAART These patients have no any logical reason to take their medication by skipping their does for few days or even for weeks. We always try to educate these patients that such a kind of medication-taking practice is wrong and may cause strong damage to their bodies as it creates an opportunity to develop the body drug-resistant virus. And if it happens in the body the final outcome would be impossible for the individual to be treated with HAART. Additionally, we educated them that if the drug-resistant virus has developed in the body this would not only affect the women but is very damaging to other HIV-positive individuals in the future.

P3: That is right. I have also seen some HIV-positive women with no interest in taking HAART daily. They tell you that HAART has a benefit which helps them to enhance healthy life and survive long. They also don't want to completely stop taking their HAART but they decide that taking HAART with making the body free from ARVs for some days is a good way of HIV treatment. Taking their HAART in such an irregular manner is more beneficial to them than they are taking HAART regularly on a daily basis. In addition to what has been said by my colleagues, there is another important factor which challenges HIV-positive women in their medication-taking practice. Those women who have a depressed mood, are unable to take their medication appropriately. I, for example, observe a depressive mood in some HIV-positive women taking HAART. when I assess their HAART-taking practice, I usually come up with these women who don't take their medication at the correct dose and right time on a daily basis. They always have very poor adherence to their medication. due to this situation, their health condition has not been as such improved as compared to those women taking their HAART as prescribed. What I have seen in general. Depression is one of the factors which affects the correct taking of HAART in HIV-positive women. Patients tell us that they are not interested to take medications at the right time when they go through a **<u>depressive</u>** mood. This is one of the reasons for some patients who missed or don't take their medications properly. In this regard, we always take much time with our HIV-positive women just to discuss the depression they have and after we finished the counseling session we sent these

women taking HAART to the psychiatric department for further investigation and better treatment.

M; What do you think is the reason for their depression? How do they tackle their depression as they are taking HAART?

P4: Yes, depression is a mental process which interferes with any kind of daily activity of human beings. To be specific, we see that some HIV-positive women develop this depression either due to the side effects of some ARVs or due to some other circumstances which surround these patients and this may be internal or external. As we all know, most females have many burdens related to economic problems, family responsibilities, and any related psychological problems. Some women tell you that this depression will disappear while they discontinue their medication. due to that to avoid their depressive mood they prefer not to take their medication or skip some doses. Therefore, the cause of HIV-positive women's depression is multi-faceted and needs to give special attention by healthcare providers. Unless these women get relief from their depression by any mechanism, the correct taking of their medication would be in question. But as a healthcare provider what I want to mention here is, not a few women taking HAART, interrupt or skip their medication due to depression and thus it is one of the facilitators of non-adherence to HAART.

P1: As healthcare providers, we always assess our patients' overall health condition, and the practice they follow for taking their HAART, and also we investigate what looks like their HAART adherence. In order to assess their general health condition we use different assessment mechanisms like clinical and immunological approaches. During the time when we make an assessment of our HIV-positive women, we identify different factors which can negatively affect the HAART-taking practice of women or can facilitate the medication-taking practice. There are many challenges we identified through the assessment of adherence to HAART in women. Depression, fear of disclosure of HIV status, lack of hope and courage to take their HAART, long waiting times at clinics due to different reasons, and lack of services at weekends are some of the challenges I have seen.

M: You have mentioned some of the challenges and facilitators of HIV-positive women which affects the correct taking of medication, would you please tell us how these challenges and facilitators affect women's HAART-taking practice?

P10: We have mentioned some of the challenges that affect the correct taking of medication in HIV-positive women. This is the major point that we should discuss here because many challenges which HIV-positive women faced while taking

their HAART can be solved through different mechanisms either through the healthcare providers, the patient herself, or family and community. In order to solve such challenges in the medication-taking practice of women, first it needs to identify the real challenges raised by the individual woman taking the HAART. Then needs further discussion to be held between the patient and healthcare providers. Then after the potential solutions to avert that specific challenge should be identified and put into practice. This is the mechanism that we apply in our clinic as a routine activity. To come to the point raised by the researchers, most of the time patients including men and female patients are interested to talk with their physician about whatever kind of health-related issues they want. Due to that while we meet our HIV-positive women when they come to our clinic during their appointment date or other time just in need of a consultation, we always assess their medication-taking practice and thus we find different challenges as well as facilitators which interfere with these women's medication-taking activities. As we interact with different many patients, we have found different issues related to that specific individual patient condition. Some women tell us about their medication-taking problems which affect their adherence to HAART. The many problems or challenges they confront while taking their medication, busy schedule and forgetting doses, religious ceremonies, taking multiple drugs and pill size are some of them. All the mentioned issues are the major challenges that affect their normal or correct medication-taking practice. This is the reality that those HIV-positive women tell us and we also observe these factors as challenges for women that make them not take their medication correctly as intended.

M: Would you please explain how these challenges affect women’s medication-taking practice?

P2: Okay, as we all know there are so many challenges or problems existing in HIV/AIDS treatment nowadays. It is clear that HIV treatment is a kind of complex issue because there are issues related to HIV-related problems which affect human beings at different individual and community levels, economic issues, issues of treatment with HAART for lifelong, and fear of disclosure of HIV status are some of them. We, as healthcare providers, notice different factors which prevent those HIV-positive women from the proper taking of HAART. From the many challenges we know, spiritual issues are common. For example, some Christian women go to religious places like monasteries just to get treatment with holy water. This is a very common issue which usually practised by many HIV-positive women. HIV-positive women who go to the monastery for their spiritual issues, always discontinue their medication taking for some days or weeks. They may not come to the clinic on the appointment date to collect their medication

timely because of their absence from their resident area. When they return home they may or may not continue their medication as they believe that they have been cured from HIV. They may stay longer at their home without taking their medication. This is the common challenge which we have appreciated as a facilitator for poor adherence to HAART. Some HIV-positive women patients come to the clinic after they have lost to follow-up their HAART for months. They don't come to the clinic [HAART clinic] on their scheduled date for refilling their medications. When we ask them why they don't attend the clinic for their medication refilling during their scheduled date, they tell us their reason as they were in a monastery for holy water. On the other side, these patients always complain about the multiple drugs they are taking. When we say multiple drugs, it doesn't mean only ARVs but these women may take other drugs for other illnesses they have. These patients may take one or two pills per day and this may not be much. But the concern they raise is they may take three, four or more drugs in addition to the ARVs they are taking. Finally, the pills they are taking might be much and thus these patients become exhausted. During this condition, there are some women patients who skip or discontinue HAART for days or months. In general, pill burden is one of the problems those patient mention as a challenge which prevent them from the correct medication-taking practice. In addition to this, as we know most females in our society are responsible for different home-related management issues. They prepare food for the whole family, they provide care for children, husbands and other elder persons within the family. She also expected to be a source of money for the expenses needed for the family. Due to this huge responsibility of women, they may go out to search for any kind of work which they are fit to do. Women are a source of money for the family. She may spend much time on her outside work she is engaged to get some money. After she return to her home there are also different home activities waiting for her and immediately she would start working there. During these different circumstances, she is always busy with her work and thus, she may forget taking of her medication timely.

P:9 As it has been discussed, forgetting to take the medication in a timely manner is one of the challenges in HIV treatment. Travel to somewhere too far from their residential area, is one of our patients' reasons for forgetting their pills to take. When they travel, they usually forget to take their pills with them and thus, until they return to their residence, they wouldn't take their pills. We always try to tell our patients in order to take their medication with them when they travel to other places and stay there.

P:8 From the many challenges mentioned, there is one issue that hasn't been raised. What are the gender-related demands which are highly related to female HIV-positive patients? Because our community give emphasis on gender-based roles

and responsibilities, those women living in the community have special responsibilities with regard to their families as well as their community. Here in our community, women are highly burdened with many roles and responsibilities which start from their home-based care and activities. Therefore, HIV-positive women have responsibilities which need to be performed in their homes. They are expected to provide special care for their children, partners and other family members in need. They have no enough time to take their medication as they are responsible and busy with multiple gender-based responsibilities.

P:3 Yes, **gender-based norms** are one major factor for women which prevents them to think about their entire health condition especially when they seek medical advice from healthcare providers. Because these women have multiple responsibilities, they have no right for their partner to go anywhere they like without their partner's permission. On the other hand, they are not volunteering to disclose their HIV status to their partner due to the consequence it will happen to them. If their partner has the knowledge that their wives have been diagnosed with HIV, they may stigmatize them due to their illness. Therefore, **gender-based roles and responsibilities** and the **fear of disclosing HIV status** are the major challenges to women's HAART adherence that need to be mentioned here. Almost three weeks back a woman with an HIV-positive result told me that she had been pregnant for three months, and she was taking her ARVs daily at the right time as prescribed by healthcare professionals because she wants to get HIV free child.

P:6 As has been mentioned, I also have noticed that some women have a challenge in taking their medication due to their **gender-based** roles and responsibilities which in our country is seen by gender imbalance between men and women. This issue is highly sensitive as it is the community's cultural role and structure. This **gender-based** role mainly affects our women by engaging them more time in their home activities and providing care for their family including their children, husbands and other old members of the family. When we observe this, it is directly or indirectly affecting those women who are taking their medication. Some women tell us that they sometimes miss their doses due to they are being busy with their multiple **gender-based** roles and responsibilities in their families.

P6: Yes, as our colleague has mentioned, the problems or challenges we identified on those women taking HAART as a facilitator to poor adherence to their

medication are many. The common challenges women faced while they are taking their medication are religious factors, pill burden and pill size, and misunderstanding about HAART are some of the challenges we identified on these women. These challenges are highly influencing negatively their medication practice. Some women raise their complaints about the pill burden they have encountered. As previously said by other healthcare providers, when we say pill burden, it doesn't mean that only related to the ARVs. These patients may take additional pills other than what they have already been taking. For example, patients may take other drugs for hypertension, diabetes mellitus, or for other chronic illnesses. During these medication-taking times, patients complain that they are taking multiple drugs, therefore, they don't afford to take them. Finally, they decide to discontinue their HAART for some time until either the number of pills they intend to take become less or to get their body some rest without ARVs. On top of this, women taking HAART still have strong fear about the medication they have been taking. They understand that taking HAART for a long time may cause different negative outcomes on their health. Though these women know that HAART is important to their life, they also perceived that if they take HAART for a long time, they would become permanently disabled. One patient told me her strong fear about side effects from HAART, that it is a long-term medication which exposed females to permanent body disfigurement... She added "We, my HIV-positive colleague and I remember a woman who took the medication for 8 years; now she becomes weak, her lower extremities are now not functional (unable to work) and she is shapeless. Though we always provide those women taking HAART with routine adherence conseling and let them get detailed information about the importance of HAART, they still listen to some rumours which make them understand HAART in the wrong way. Actually, such poor understanding or misperception about HAART becomes decreased but the major thing that we should know is, still this challenge is existing in the practice of women medication taking. What is more, **fear of disclosure of HIV status** is another major challenge forr women taking HAART. This stigma and discrimination starts from the family as we have seen in many patients. Supports from husbands, children and employers have good results in HIV treatment. If a woman has no support from her husband and from her family she will feel as if she is stigmatized and this will result in poor adherence to her medications.

P1: We observe that many women skip or discontinue their medication because their family members haven't given them any kind of support rather they [family members] don't eat, drink and talk with these women. During this circumstance, those women have a feeling of stigma, and discrimination and thus they don't want to take their HAART at the right time. They become disappointed with what

they have seen from their family members about them. On top of this, most HIV-positive women **don't like to expose their HIV status even** to their husbands because of the fear of the stigma that might be occurred by their husbands. Unless those women tell their HIV status to other individuals whom they trust, they will have no support in the treatment they have received from health institutions. The major reason for **not disclosing their HIV status** which mostly mention by HIV-positive women is stigma and discrimination. Women taking HAART usually tell us that have a fear of noticing their HIV status with others; if somebody knows these patients' HIV status they will be stigmatized by different individuals including their family and friends. Sometimes these women change the clinic that they have used before to some other one due to the fear of not being seen by others in that clinic.

P4: Yes, fear of disclosing HIV status is one of the major problems in our society. When I asked my patient during her clinic appointment day in this clinic, whether she follows the proper medication taking the time or not… she replied to me that she has been working in others' homes by baking ' Injera' [local Ethiopian staple food] the whole day. She has two children [who are HIV negative]; she is the only caretaker of them. She doesn't take her pills properly because she fears employers that if they are aware of her HIV status and medication taking, they would stop her from working in their home. She only takes her medications when her employers will go away from her and she will be alone.

P9: In our community, more women with HIV hide their status fearing to be deserted by their male partners after disclosing their HIV status. Even though they have been taking their antiretroviral medications, they may not tell their male partner and what they do is they will take their medications to hide from their partner. Due to this, these women may not take their medication daily and even will discontinue for some time until they get a convenient time to take their medications. There are many women taking HAART who tell us about their fear of disclosing their HIV status even to their male partner. They usually tell us that if they are detected by others they will get neglected. If an employer knows that an HIV-positive woman is in his organization, he will immediately fire her from the organisation. I remember a woman with HIV who shared her history like this; "If you are a private employee, you will be fired from your job as soon as the employers know your HIV status. Those HIV-positive women who work for such private employer organisations, will not take their medication during their proper time as they will be seen by employers when they swallow their medications. Similarly, these women may have no permission during their hospital appointment and due to this they will not collect their medication timely. I know

one young lady in our community who lost her job at a flower farm share company after her boss noticed that she had been taking her antiretroviral medications. Therefore, disclosing your status may bring difficulties in your overall life and that is why in our community most people do not like to disclose their HIV status."

P5: Yes, as it has been said because of the fear of stigma and discrimination some women taking HAART don't like to tell their HIV status to anyone else including their partner. This condition becomes highly significant and creates serious problems when we come to those HIV-positive women taking HAART. To avoid their identification by others in their residential area some HIV-positive individuals prefer to follow their treatment [HAART] at another hospital like from Yirgalem [a town about 42 km far] when they get access to it in Hawassa [a town where one study hospital found], and vice versa. After some time, they will be challenged [lack of transportation, distance etc.] while they try to re-fill their medications during the scheduled day. Patients in such circumstances will not attend their treatment properly.

P10: The other problem which affects most HIV-positive women not taking their medication as intended and prescribed is economic. As mentioned by many of our colleagues, women who have no money may not attend the HAART clinic during the clinic appointment date because they may not have money for transportation. There are women taking HAART they have no money even to pay their transport cost to come to the HAART clinic. If these women taking HAART have no money will not be able to collect their medication from the HAART clinic during their appointment. There are also women with HIV who have no money for buying food. If they don't have food before their medication they don't want to take their medication with an empty stomach. Therefore, a lack of money for transportation and buying food may lead these HIV women not to take their medication appropriately. Finally, these women wouldn't be adhere to their HAART because of the money they lack. We hear from some HIV–positive women that they are not able to be hired by the organisation or private homeowners because of their HIV-positive status, and this unemployment makes patients unable to get access to food and this also enforces them to travel too far just to seek any work opportunity. Finally, these women don't get money for their daily expenses and their transportation costs to the hospital.

P2: One woman taking HAART told me that "It is unquestionable that HIV is very, very serious in many conditions like it affects your health, social and economic status. From the very beginning, it will expose you to many different diseases as it kills your white blood cells which make your body unable to fight diseases. If

the virus [HIV] get entered your body you should eat good food to make your body strong but most of us do not have sufficient money to buy and eat these good foods; if you take me, I sometimes skip my doses because I don't want to take my medication with an empty stomach." Some HIV-positive women think that if you have no foods like meat, egg and milk, it is better to skip some doses or stop taking HAART. This is the wrong idea and thus we always try to educate our women and men taking HAART through the counseling sessions provided by our clinic. On the other hand, females are not well educated and usually they are economic dependant. In most cases, females are expected to stay home, to provide care for children and old family members and due to this; they have no access to better education and job opportunities. As most females have no better work for money, they always struggle to get money by engaging themselves in trivial activities and labour work. Since they don't get enough money for her, her children and her family, she tries to cover expenses by employing herself in daily labour. If there is no money to cover the woman and her family expenses, she may not take her medication properly and finally, she will have poor adherence to her medication [HAART]. As has been mentioned now, pill burden, as well as pill size, is one of the reasons mentioned always by HIV-positive women for not taking their medication correctly and the right dose at the right time. Many of our patients were complaining about the bulk number of pills they were taking and due to that some of them discontinued taking their daily medication. But these days, these problems are not an issue for most patients. What we have understood from this, one fixed pill medication is highly helpful to our patients and makes them adhere to their HAART. The number of pills they have taken is the reason that makes discomfort in women in the medication-taking practice. In addition to that, they also tell us the size of ARVs is big to swallow and creates inconvenience in women during the taking of their medication. Due to this, some women skip some doses just to avoid and forget the inconvenience which would be created by the big size of ARVs. They also tell us they are taking many pills together in one day though the ARVs tablet would be one or two per day. But there are also some other drugs prescribed by their physicians for other illnesses therefore they are intended to take ARVs and drugs for other illnesses per day. This creates a high burden from taking many pills and thus they sometimes inforced to skip some doses and even interrupt their HAART for days or weeks.

P4: As mentioned by others what I sometimes observe in these patients, is the pill burden and pill size of the medication they are taking create discomfort on them while taking their medication. ARVs pill burden is not currently a major problem for our patients. Nowadays, most ARV drugs are prepared as a fixed (Two or three different drugs are prepared in one fixed tablet) dose formulation. Actually, there are some patients who complain of pill burden because they are taking

ARVs with some other prescribed drugs for other illnesses. If those patients take drugs for other chronic illnesses like diabetes, hypertension and others, they usually complain about the pills' burden and thus, they sometimes skip some doses or discontinue ARVs to avoid such burden due to multiple pills.

P9: Due to different reasons some women with HIV complain that they are not as such as other individuals without HIV. They tell you that because of the long time taking HAART, and the lack of drugs to cure HIV is disappointing them and due to this, they considered themselves as individuals without any hope in the future. Due to this thinking some of the women patients with HIV are not in a position to take their medication properly.

P7: During our adherence conseling session, many women taking HAART tell us many things with regard to their medication taking. There are things or situations which facilitate their medication taking properly. On the other hand, there are things to be mentioned by those women as a barrier to taking their medication in the right dose and at the right time. As recently has been said you sometimes get women with a hopeless future as she has no supporter in their medication-taking practice. Some of the women who mention these have husbands but their husbands don't support them rather they make these women stigmatized.

M: Do you have any additional things to be mentioned especially about challenges which interfere with the medication-taking practices of HIV-positive women?

P6: Just to add some points to what has been said by my colleagues, some HIV-positive women have always told us about the working day and hours of the HAART clinics. As we all know our clinics are working during office hours which don't include other weekends. Due to that some women tell us that they miss their pills because no drug was at their hand during the medication-taking time. the reason that they had no drug at their hand as they were unable to come to the clinic on the clinic appointment date to collect their medication because of their work that is being done through weekdays only. There is no other option for such women taking HAART to visit HAART clinics just to collect their monthly medication other than at weekends. Due to the inconvenience of the HAART clinic's working day for some HIV-positive women, they may not collect their medication from the clinic timely and thus it would affect the proper medication-taking practice of women taking HAART. but on the other hand, we provide those women taking HAART the needed services as soon as possible. we provide our service to our patients as soon as possible. We don't want them to stay a long

time in the clinic. If patients have no other medical conditions which need further examinations like laboratory services, they will not stay a long time at the clinic.

P8: There are times that patients need to have investigations, for example, laboratory, psychiatric, and gynaecological when they come to the clinic on the scheduled date to collect their medication and thus they may stay long hours in the hospital. However, some patients don't stay here long and leave the hospital without refilling their medication.

P1: I also agree with what has been said about the weekends service. HAART service provision is only given to patients from Monday through Friday but due to the nature of their work, there are some HIV positive women who don't have free time during these days and thus, they may not come to the clinic and collect their medication timely.

P9: Yes, this problem is the issue of some HIV-positive women taking HAART. I know some women who missed their medication because of the absence of ARVs at their hands due to the heavy work they have from Monday through Friday. During these working days, their employer is not happy to give permission to their workers and thus these women taking HAART don't like to ask permission to go to the HAART clinic. These women will only have free time during weekends to collect their medication but during these days the HAART clinics are not open to provide services for their patients. We are working only from Monday through Friday. But a number of patients want to come and collect their medication from our clinic only during weekends due to different reasons. As these patients have no access to collect their medication during their convenient time at weekends, they would miss some doses when their medication run out with no timely refilling.

M: How do you describe the HIV-positive women taking HAART adherence to their medication?

P4: Okay, it is possible to talk about the adherence level of HIV-positive women who are following their HIV treatment in our clinic simply by saying something like good or bad. But the good thing here is we healthcare providers know that one reality which is the majority of HIV-positive women have good adherence to their HAART. However, this is not enough for the discussion here because we want all HIV-positive women to have good adherence to HAART. Still, there are so many HIV-positive women who have poor adherence to their HAART because of different reasons. Therefore, the important thing here is, yes, the majority of the

HIV-positive women are taking their medication properly and thus they have good adherence to their medication. On the contrary, there are also many HIV-positive women who are not taking their medication properly which means they are not taking the right dose at the right time. To conclude my understanding in this regard, not a few HIV-positive women have poor adherence to their HAART. We have already discussed many barriers to HIV-positive women's adherence to HAART.

P2: When comparing those HIV-positive women with good adherence to HAART with those HIV-positive women with poor adherence, it is easily understood these issues in our day-to-day interaction with our patients. Therefore, it is impossible to put in terms of a number of good adherence and poor adherence but it is possible to know that the majority of HIV women have good adherence to their HAART but some of (not few) the HIV-positive women do still not have good adherence to HAART. But this should be seen as alarming that we still work hard on those women taking HAART with poor adherence. As we explained and discussed in detail there are factors which determine HIV-positive women to have good or poor adherence to HAART. The government should work strongly to solve some kind of barriers which affects women’s adherence to HAART. On the other hand, research like this should also be strengthened to identify the real problems and put some strategies to facilitate HAART adherence.

M: You have mentioned some of the barriers which affect women's adherence to HAART. What do you think are the facilitators of adherence that you have seen in HIV-positive women taking HAART?

P1: Okay, when we observe our HIV-positive female patients' adherence, as has been mentioned earlier, the majority of females are having good adherence to their medication. There are many reasons to be mentioned here as facilitators of adherence to HAART. Some of the facilitators to good adherence in HIV-positive women are responsible for family and caring for children and understanding the benefit of HAART, and social and family support are very important ones. As we all know most women are mothers and have children and thus they are responsible to provide care for their children. Similarly, women in our country are the home managers, they are also responsible to the family as a whole. She is required to perform each and every needed activity of the home and she is also the one who provides care for her husband and other family members. Due to this, they [HIV-positive women] are more responsible to have better health conditions. They want to provide maternal care for their children until their adulthood stage. They also want to see their children's future fate after they

have completed their university education so that they want to have good health conditions and have a plan to live longer. Thus, these HIV-positive women take their medication properly which means the right dose at the right time. The other facilitator of adherence which I know these patients is the understanding they have towards the HAART. Some HIV-positive women have a good perception of the importance of ARV drugs. They always tell us the benefit they have gotten from the medication they have been taking since the time they started. They are healthy and take their medication without any missing or interruption. They may be challenged by some kind of side effects occurred by HAART but they don't want to interrupt their medication due to these side effects because they know what will happen to them when they interrupt their medication. Many HIV-positive women have better knowledge and understanding of the benefit of HAART and good adherence. These patients collect their medications from the HAART clinic on time and take them appropriately on a daily basis.

P10: Yes, as it has been mentioned, many HIV-positive women have better knowledge of the benefit they get from HAART which they have been taking currently. Due to that they don't miss any dose and give great attention to their adherence to HAART. In addition to their knowledge about the benefit of HAART, those HIV-positive women taking HAART are always responsible to take care of their children until they grow up to adulthood stage. Unless these women have a healthy life, their children would be in danger. Therefore, they keep themselves healthy by taking their medication properly this is not only for them but they strictly follow their treatment for the sake of their children and family.

P8: Many female patients who have been taking their medication, tell us that they are responsible to manage their home which means the entire family members including their children, husbands, mothers, fathers or others. Therefore, these women think that if they are not healthy and not able to work even for a moment, there are many individuals who would be affected. Due to that, they are taking major action which would help them to have good adherence to their medication. Actually, there are also some women who have no detailed understanding of their responsibility and expectations from other individuals. These women taking HAART are not responsible for themselves as well as other family members. To finalize my opinion in this regard, because of the responsibility for caring for children and family is one of the facilitators to adherence which I have seen in HIV-positive women on HAART. In our experience, those HIV-positive women who have children are more responsible to take their medications properly when compared with HIV-positive women with no children. The other thing which has crucial importance in adherence of HIV-positive women is the support they get

from their family and other social interactions. Some women have either psychological, material, or other social support provided by their family or other individuals in the community. Those HIV-positive women who get support from their male partner are psychologically stable and take their medication correctly at the right time. For example, their family members help these HIV-positive women to take their medication correctly by reminding them of their medication. Family members also support these patients psychologically as they show that they are with these women at any time for any kind of support. Some women also get some economic support from their family members while taking their medications. Therefore, their family and social support are found to be one of the facilitators to have good adherence in HIV-positive women following their treatment.

P2: Just to add one point, especially on patients expereincing on their improved health condition due to the medication they have been taking. Many HIV-positive women had developed different opportunistic infections (OIs) before they diagnosed with HIV and even after they knew their HIV positive status due to fear of starting HAART. But after they have started taking HAART, a significant improvement of health condition due to HAART makes them stronger and to take their medication properely as prescribed by healthcare providers.

P6: The adherence counselling that we give to our women patients has very much importance as we revealed by the outcome we get from our patients. Those HIV-positive women tell us that they get much information and knowledge about the medication they have taken and thus they properly take their medication as intended i.e., the right dose on a daily basis. Therefore, continuous adherence counseling sessions could bring many changes in women to have better knowledge especially those who hadn’t good adherence to HAART. After having better knowledge about the benefit of HAART and the importance of adherence to HAART, these previously non-**adherent women could have good adherence to their medication. Therefore, adherence conseling which is provided by healthcare providers has an important effect in facilitating HIV**-positive women's adherence to HAART.

P3: Like that of ART adherence counseling, the information and education which is given by adherence-supporting group members have changed the understanding of some of the HIV-positive women regarding ART benefits and the importance of adherence to their medication. Some women taking ART tell us the support they get from the adherence supporting group helps them take their medication in the

right manner. It also helps these women to be psychologically stable and live a healthy life. One woman who is on HAART told me that she was interrupted by her medication for two months without telling healthcare providers and her sister. But those HIV-positive women who are a member of the club shared their experiences with her about their medication-taking practice, and then she started to take her medication because of their support therefore, her health condition has improved; now she is okay with everything.

P9: Oh, the importance of adherence supporters group is significantly high. These group members are all of them HIV-positive females who have good adherence to their medication. They provide their good adherence experience and adherence conseling through peers. Some women who don't want to tell us the information about their medication and treatment, sometimes tell the reality to their peer adherence conseling supporters. These adherence supporters provide HIV-positive women with their overall life experience regarding the treatment of HIV. Then most of the women taking HAART have got best life experience and due to that they start to take their medication properly and finally, they would have good adherence to their medication.

M: You have discussed some of the barriers to HAART adherence which affect the HIV-positive women taking HAART. Would you please add more in this regard?

P5: These days the formulation of HIV medication has improved and thus it is easy for patients. Previously patients had a complaint about the medication they took because they were taking many drugs two times a day but now it has been changed to one fixed tablet. These fixed tablets have prepared in the form of two or three drugs fixed together in one pill. This drug formulation has reduced the burden and fatigue of HIV-positive women due to multiple drugs with many pills to be taken two times a day. These days the drug formulation is attractive and the frequency of taking these drugs is reduced to a single tablet per day. This condition makes women patients take their medication easily and have good adherence to their medication.

P1: Yes, currently many patients take their pills one time a day. But there are also some other HIV-positive women who need to take their medication two times a day. When comparing these two groups of patients [those who take pills one time a day with those who take two times a day], those who take their pills two times a day have discomfort as the increased frequency of taking pills creates a pill burden and additional time in a day to take pills.

P6: Taking only one pill per day is very helpful because some women were not taking their medication properly previously due to the burden and fatigue they had due to multiple drugs. But as mentioned recently by our colleagues, the one-pill drug formulation has changed some women's medication-taking practice as it helps them to take their medication properly because it is easy to take one pill per day. Therefore, one pill per day drug formulation facilitates women's HAART adherence by making drugs be taken easily and comfortably.

P3: As we mentioned previously, some of the HIV-positive women may skip their doses due to forgetting as they are so busy with their different activities. On the other hand some HIV-positive women on this regard, they are not missing their doses because they have been using some kind of reminders to take their medication at the right time. A woman who works in the flower farm industry told me that she hadn't taken her medication properly. Before three months she missed some doses as she was busy with her work and forgot to take her medication at the right time. However, she has used her mobile alarm for reminding the medication taking time, therefore she hasn't missed any dose since then.

P4: This is a very important issue. Because many women taking HAART sometimes miss their doses as they are either busy with their home and workplace activities or they are asleep during mediation taking time. By the way, this is very common in many women, if they are busy or fall asleep they usually miss some doses repeatedly as they have no reminder during their medication taking time. Actually, there are women who get such reminders from their family, colleagues or other individuals. Similarly, some women use their mobile phones or clock alarm as a reminder of their medication-taking time. In general, those HIV-positive women who apply some reminders like setting alarms on phones or watches are taking their medication properly at the right time.

P9: We understand that patients using any kind of reminder in their medication-taking practice are in good adherence status to their HAART. We always advise our patients to have a reminder if they are at work which might make them busy and forget to take their medications. Some patients tell us that they are using either a watch or mobile phone alarm to remind them to take their medications on time.

P10: As I have seen in my encounter, those HIV-positive women who are using a kind of electronic reminder, especially setting their mobile phone, and clock alarm to call or vibrate at the right time that patients intended to take. As this is a method which helps HIV-positive women to remind their medication time. Therefore, missing doses during the medication time is not a problem for those who are

using the method. Currently, many HIV-positive women use medication-taking reminders other than any supporting person. Therefore, these women use the alarms of their mobile phones and watch as a reminder method because they want to take their medication appropriately at the right time as prescribed by healthcare providers.

M: Okay, you have mentioned different issues related to HAART adherence of HIV-positive women. The different issues you mentioned are the challenges which prevent HIV-positive women from the proper taking medication on a daily basis. Therefore, the barrier which negatively affects women's medication adherence are busy schedules and forgetting doses, fear of HIV status identification, side effects, absence of social and family support, religious issues and economic constraints. To the contrary, you have mentioned also the facilitators that you have seen on your patients and these include understanding HIV-positive women on the benefit of HAART, drug formulation (single pill per day) and drug taking frequency and so on.

Do you have any issues or ideas that you want to add?

We have finished our FGDs. I thank you very much for taking the time to participate and for the valuable discussion you made.

END OF INTERVIEW-116 minutes transcribed.

3

Verbatim transcribed-Focus Group Discussion-3

<u>Transcription results:</u>

M: What are the perceptions of your experiences about HAART adherence of women who are HIV positive?

P4: As a healthcare provider of hospital X HAART clinic, I have experience with different practices regarding those HIV patients, especially those female HIV-positive patients. We know that throughout the world there are more HIV-infected female patients than HIV-infected male patients. This is due to different reasons like biological issues, male dominancy over females, cultural factors, economic factors and the like. When we come to those developing countries in Africa like

our country, Ethiopia, there are more female individuals who are HIV positive than those male HIV positive individuals. Therefore, this is crucial and important research as it focuses on this segment of the population which means female HIV-positive patients. Since females are the backbone of the family and community, if females are healthy, indirectly there is a healthy family and society. There are many HIV-positive females who are following their HAART in our clinic. The number of female patients who are following their HIV treatment is higher than their counterparts. When we talk about the adherence of those female patients to their medication, it needs deep discussion on each related issue. Because the term adherence by itself is a broad concept. There are two kinds of adherence, these are good and poor/non-adherence, therefore, we need to talk by exploring these adherence types related to female patients.

M: You have mentioned there are two types of HAART adherence. Would you please elaborate more on the concept of good and non-adherence?

P7: Sure, if those HIV-positive women on HAART taking their medications properly during the right time and the right dose without skipping any dose and interruption are said to be they have good adherence. On the other hand, if HIV-positive women on HAART are not taking the prescribed ARV medication according to the order of the physician, which means the right dose at the right time, they are said to be they have poor adherence to their medication.

P5: As a healthcare provider when we explain the concept of 'good adherence' to HAART, it means the readiness and capability of patients to follow health-related advice given by healthcare providers during counseling sessions, take ARVs as intended which means the right dose at the right time, attend clinic appointments for the timely collection of their medication and adherence assessment.

P6: Actually it has already been explained just to add some points, it is the visiting of health care facilities (HAART clinics) as per the given appointment by health care providers and consistent collection of antiretroviral medications from the pharmacy at prescribed intervals. When we say 'poor adherence', it means that the patient is not keeping the HAART clinic appointments and not taking the prescribed medications according to the professionals' advice like the right dose at the right time permanently.

M: Anyone who wants to add to this issue?

P2: Okay. All the explanations described by other professionals are almost similar and correct, but when we talk about adherence, it requires informed consent

(agreement) and participation from the patient in care and treatment. The patient should agree to follow the instructions of the health care provider about the given HIV medication. If the patient is not ready to follow her treatment as per the given schedule and prescribed medication the consequence will be poor adherence to ART.

M: How do you think HIV-positive women on HAART do, generally speaking, in terms of adherence to their medication?

P9: There are female patients who can tell you about the concept of good and poor adherence if you discuss with them their adherence and other related issues during their scheduled clinic visit. When you go deep during the discussion, they will tell you that they have not taken their medications as per the prescription. If you listen to them what they want to talk to you, they will give you the different reasons for not taking their medication properly. Whether they are educated or not, they will have their own reasons which prevent them from taking their drugs. There are many HIV-positive women taking HAART who tell us that they are sometimes in trouble taking their routine medication. They mention that they have some challenges and thus they either skip some doses of HAART or discontinued it for a short or long time. It is well known that most women are very busy with their home activities. Actually, these women may have other work at some organisation or domestic houses just to get some money for their own or their family support. due to that busy schedule, they usually forget to take their medication at the right time. Women are caregivers you know? They are highly responsible for themselves as well as for their family members including children, husbands and old age people. To perform these all activities they are struggling the whole day and night. This is the issue of the majority of women whether they have HIV or not. Again, the other worst scenario with regard to females is most women are not well educated because of their cultural influence, family or husband influence. Though they have some kind of work, which is it small or big, it would be tiresome work and thus they will engage in a laborious activity. Because of their less education, they are not professional which is relatively not a king of labour work. Just to summarise my opinion, women in general, are highly involved in their home activities as they are taking care of their entire family including husbands and children. Thus, they forget their medication as they become tired and fall asleep during the medication-taking time.

P1: Though patients have an interest to take their medication there are times when have difficulties which prevent them from taking their pill at the right time. One patient told me that she was first staying with her husband's sisters at one home.

After they noticed that she had HIV, they didn’t want her to live with them, they started throwing provocative words, and they started laughing at her. Finally, she left home and now she gets support only from her friends. She doesn’t take her medication daily because she has no money to buy food.

P10: Many patients, especially females told me that adherence to HAART is not easy for them to fully follow the medication instructions and other advice given by the physician. Their most common reason was being too busy or being absorbed in working at their home and they forget to take their medication timely. This condition is more serious, especially in housewife patients as they spend most of their time caring for children, cooking food for the family and performing other activities at home. This is one of the problems which inhibit female patients from taking their medication properly as per the physician's prescription, and finally, it may lead them to have poor adherence. In contrast, some other female patients have been taking their medication properly as prescribed by the physician. I remember that one of my female patients said "Taking my ARV therapy will make me feel good; I know that I will not be cured, but it will help me to be healthy and live longer."

P3: As we all know in our country, females’ position within society, commonly obedient to men, greatly increases their individual vulnerability to HIV and AIDS. In our country, cultural and ethnic beliefs, taboos and myths could place females in extremely vulnerable conditions. Furthermore, poverty increases HIV and AIDS vulnerability; the epidemic itself increases poverty among infected people, their families and communities. From such challenges, we can say that female patients would be influenced and could not visit health institutions for their health check-ups and for collecting their medication which results in a loss to follow on their ARV treatment and finally their adherence becomes questionable. Unless the government with healthcare providers and other stakeholders work hard on this challenging area.

P8: As mentioned by our colleagues, forgetting doses and being busy are the common challenges which have been mentioned by some HIV-positive women. These HIV-positive women tell us different issues as reasons for forgetting their doses during their routine life. Some HIV-positive women tell us they sometimes forget their medication because of their personal, family and other social issues.
A woman told me “ I went to Moyale to bring some clothes to sell in the centre of the country like Hawassa and Addis Ababa. I forgot to take my daily pills with me, therefore, I didn’t take my pills for five days.” On the other hand, a busy schedule is another common problem for HIV-positive women. Most women are highly

responsible for their families. They prepare food for their children, husbands and other family members. During their engagement in-home activities, they may forget to take their medication and thus the right time for taking their medication would pass as they are busy with home activities. But there are also some other reasons for women patients not to take their medication properly. Some HIV-positive women don't want to take their medication with their religious-related issues. This is also another common factor mentioned by HIV-positive women. There are HIV-positive women who discontinue their medication while they are under their religious rituals. They usually tell us that if they are in a religious formality, they are not required to take their medication because of the doctrine which forbids them not to take any scientific or local healers made medication. In addition to that, fasting is also related to the above doctrine. If individuals are on fasting during the religious fasting seasons, they are expected not to take any medications other than some kind of food and water. During their fasting time, they are highly expected to pray rather than take any kind of medication. Therefore, if an HIV-positive woman taking HAART, is on such religious fasting, she doesn't take her ARVs because of the doctrine they must obey. Due to this, some HIV-positive women skip their doses or discontinue their medication for weeks or months.

P6: Some female patients are infected with HIV, which made them see themselves as being different from others. For example, I remember that one female patient said "I'm no longer a healthy person; I am suffering from a disease that has no cure... no remedy. Taking HIV medications is not a guarantee preventing me from developing different infections, therefore, I have no hope in the future. Only God will help me to cure my disease, otherwise, nothing will be changed by the treatment". On the contrary, there are also some other female patients who have better knowledge about opportunistic infections. They can tell you what opportunistic infections mean to them and when patients would develop these infections. Even they understand their severity unless they have prevented from appearing by increasing one's immune status through treatment and improving lifestyle.

M: You have mentioned especially being busy and forgetting doses due to some reasons are the common barriers to HIV-positive women's adherence. Would you please tell me more about HIV-positive women's adherence to their HAART?

P10: Many HIV-positive women on HAART complain that they still hide their medication from their families and others because they are afraid of the social stigma. They don't like to disclose their HIV status because they will lose their

business if their community and family know their HIV status. That is also one reason why many patients take their medication in a hidden way either before or after the medication taking time and even sometimes they may not take their medication totally if the situation is not good for them. Sadly, it is challenging for them to take their medications on time every day while they are with their family as well as to keep their HAART clinic appointments due to the fear of their HIV status identification by others.

P3; Many female patients, especially housewives don’t want to tell their HIV status to their husbands due to the fear of possible social stigma and also fear of their marriage break which will occur after husbands have knowledge of their wives’ HIV status. Many female patients are not well educated and have no job for income; they are economically dependent on their male partners. If their husbands dismiss them from their homes due to their HIV status, they don’t get money which results to affect their life as well as their children’s life. Due to this, they prefer to keep their HIV status and ARV medications secret.

P5: Some HIV-positive women, who have husbands, due to the fear of stigma and discrimination, don’t like to disclose their HIV status to their husbands. If they tell their HIV status to their husband, he will immediately divorce them and may take them out of their home and will be exposed to other problems. Due to fear of such a situation, they prefer to keep their status and treatment secret. Thus, they will not take their medication properly the right dose at the right time and finally, they will have poor adherence to their treatment.

P8: During their visit to our clinic, many HIV-positive women tell us about their fear of identification of their HIV status. In my observation, the stigma will occur, especially in female patients by their family members, friends and other people at their workplace. One woman patient, who is a mother of three children, told me that she had stopped socializing with her neighbours due to their reactions to her children having HIV.

P9: As it is already said, some of them are taking their medication properly and others are not taking their medication properly due to the mentioned reasons. Among the many reasons they tell you about not taking their drug are the side effect, fear of disclosing HIV status just to avoid stigma and discrimination, absence of family or husband support in taking of the ARVs, and the high number of pills are the reasons patients raised most while they come to our HAART clinic. But when we provide them with the information about their meditation and adherence which need to be known by our patients, we give special attention especially to women due to their very nature of them. Most of the time those females in our nation are not as such given support from the entire community as

like that of males. There are different cultural beliefs that give a special value to males than females. This situation hinders females from getting better education and better economic activities. When we see this situation with regard to the treatment of HIV, though many women are infected with HIV in our country, those who access the treatment are relatively fewer. When we observe that HIV-positive women who follow their HIV treatment in healthcare facilities have different challenges which make them not take their medication at the right time due to ARVs side effects, pill burden, lack of support from family and so on. There are patients who strongly complain that HAART has more side defects in women than in men as compared to its benefit. They [some women] tell you that their body shape has been changed badly due to the ARVs they are taking.

M: You are telling us some of the challenges which negatively influence HIV-positive women's adherence to their medication. Would you please explain this more?

P7: Some patients raise complaints related to side effects. They tell us that they have developed different side effects like diarrhoea, abdominal cramp, skin rash, nausea, and vomiting. Though we advised our patients not to discontinue, or skip any dose due to some temporary side effects, some of them still don't like to continue their medication after they have developed some of the mentioned side effects due to this they are unable to adhere to their HAART. In general, some HIV-positive women discontinue or skip some of their doses due to the HAART medication.

P5: Yes as it has been said, the issue of ARVs side effects is the major means of HIV-positive women taking HAART to skip some doses for some time like from some days to weeks or months. They tell you a lot of things related to the side effect they have experienced or they have been informed by another person. The common side effect they usually raised when they come to our clinic is continuous headache, dizziness, abnormal distribution of body fat like the accumulation of body fat at their abdomen or back of their neck and the removal of fat from their face and back, abdominal cramp, and nausea. In this regard we as healthcare providers, especially those we are working in the HAART clinic always think about our patients' health; this is our responsibility i.e., professional responsibility you know? Therefore, we first counsel our patients on how to start the medication and how to properly take their medication. This counselling also includes how they adhere to their medication and if not adhered to their

medication what will be the consequences and what expects from them are discussed. Then after they are also having a chance to participate in the decision which will be made on the initiation of the ARVs. After they have already come up with consequences we advised them to start their medication. The major issue here is the explanation of HAART benefits and the expected side effects from the ARV drugs are given special attention by the healthcare workers and thus these all issues are briefly discussed with the patient. Finally, they start taking the medications prescribed. Then after they follow their schedule to visit the HAART clinic just to check for any health-related issues, and refill their medication. The major concept here is we always have to follow the status of our HIV women's adherence to their medication, and we always counsel them not to skip some doses or not to discontinue their medication due to side effects as the majority of them will disappear after few weeks or months but still, there are HIV positive women who skip or discontinue their medication due to side effects.

P4: The other issue which is always complained about by HIV-positive women is the size of pills they have been taking. They tell us that the number of pills especially the ARV is reduced but the size of ARVs is very big and they have discomfort with that. Some women tell that their previous medication especially the number of pills and the number of medication taking frequency per day is now reduced except for some of those patients taking second-line regimens. But the pill size is not convenient as it is very big and due to that some women tell us to skip their doses just to avoid and forget the inconvenience with the big pills. Many HIV-positive women have understood the benefit they get from their daily taken medication, but they don’t like taking these pills continuously on a daily basis due to the discomfort creates by the big size of the pills.

P2: Sometimes, they tell you that it is challenging for them to take ARVs every day of their life, especially when taking these pills with other kinds of pills and the daily intake that is required to be coupled with other additional drugs for other illnesses.

P1: Yes I have also experience with what has been said now. Some women patients do not like the sizes of some ARVs. Due to that, they have complaints about the medication they have been taking currently. A woman who doesn't take her medication properly told me: "Okay, first, of course, would be the size of pills; because the HAART drugs are not like other illness drugs; due to that I take them with discomfort… and skip them, meaning I do that sometimes. They are so big to swallow as well as they are not convenient to hide in some places just not to be seen by other individuals. They are all big pills and easily detectable by somebody else when I put them in my bag or at home."

P3: There are also some HIV-positive women taking HAART who raise some strange issues as a reason for not taking their medication properly. There are some HIV-positive women who don't like to take their medication [ARVs] daily. We always tell our patients to take their ARV medication daily at the right time but some of them still have heard and are convinced by some rumours that since HIV is not curable by HAART, no need of taking HAART daily for not curable virus; taking of HAART every other day is enough to our body to control the virus.

P7: As my colleague said, there are patients both women and men who don't like to take their routine ARVs as recommended. Some of these patients tell you that they have been taking their medication two or three times a week for an unacceptable reason. The reason they raise for not taking their medication properly like every two or three days a week is, the virus is not curable by any kind of drug including HAART. We have observed that those HIV positive women who had diagnosed with some kind of psychiatric problems, for example, depression; usually don't take their madication as prescribed by the healthcare providers because of the depressive mood they have. Thus, the function of HAART is only to suppress the virus; due to that to avoid the potential occurrence of depression they prefer to take their pills by skipping them. Some women tell us that they are in depression since the time they started the medication. Thus, they discontinue their medication for weeks or months. Those women who tell us they have depression during the taking of their medication, have usually poor adherence to their HAART. As health care providers, when we have such kinds of women patients, we provide them with appropriate conseling and send them to the Psychiatric clinic for further investigation and treatment.

P6: When they have such a mental condition, meaning depression, we always give them proper medical advice and send them to the hospital psychiatric department for further investigation and treatment. We give special attention to patients with

depression as this medical condition is the means of patients' poor adherence to HAART.

M: You have mentioned some common barriers to HAART adherence of HIV-positive women taking HAART. Would you please tell me more about any barriers to women's adherence to HAART?

P2: As anyone knows, in the majority of cases the role and responsibility of women is not an easy issue rather it is highly important within the family as well as the community. In my opinion, most women have different roles, for example, they carry double the burden of raising children, performing household responsibilities and earning an income for their family. Look! If these women become infected with HIV and sick, you can imagine what will happen to their children and family. Things will be changed and the children and family will also be faced challenges. That is not the only issue, if we think that these female patients have such a burden, how do they follow their HIV treatment? Can you imagine this? Really, this is most patients' problem, which hinders them from taking their ARV (antiretroviral) and other medications properly as prescribed by health professionals.

P10: As healthcare providers, we know that the role of women is multi-dimensional. They play a crucial role in a family as well as in a community. Females are mothers, workers, managers, caregivers and the like. It is not easy to explain their role in simple words. Especially, in countries like ours (Ethiopia), they are economic-dependant and have no access to education. Women are under the control of males; this makes them not decide about their health on their own

P1: Actually, the most common challenges which need to be mentioned here are well discussed. But just to add a few points on this issue, there is also one point that is a common issue related to women's adherence. Some women raised an issue which is difficult for them in their medication-taking practice. They always ask us why the HAAT clinic not working weekends, and they tell us that during the weekday they are too busy with their work to come to our clinic. Therefore, they are unable to collect their medication during the weekdays. This may be a reason to discontinue their medication as they are working at home or some other place and due to that they wouldn't come to the clinic to collect their medication. Since they haven't collected their medication on time they may have medication at their hand and thus they have no medication to take.

P4: There are untouched areas about HIV-positive women's HAART adherence. Many HIV-positive women patients live alone or live with their families but receive

little or no care and help from their families. We have several patients who are living by themselves because their families try to abandon them or people from other towns move to this town or nearby so they have no family support. It is quite difficult for them, if they forget their pills or oversleep, no one will remind them. They don't know what to do, just try to remember to take the pills by themselves if not possible they will not take their pills at all. So that, due to their economic problem and lack of social support, these patients will not have good adherence and at the end of the day the probability of developing drug resistance is very high.

P9: Yes this is also my opinion, I have always had women in our clinic with such ideas. There are patients who tell us that the scheduled date given by the clinic to refill their medications is not comfortable to them because they are labourers, and have no time from Monday through Saturday unless they have been given appointments on Sunday [work free day]. I remember two women who skipped repeatedly their scheduled date to refill their ARV medications due to being unable to come to the clinic during weekdays.

Many issues, especially those related to the factors which impede HIV-positive women from properly taking medication have been pointed out. Some patients are also complaining about the time they stay at the clinic until they get their pills. There are patients who are irritable and they don't want to stay even for 15 minutes at the clinic. Such patients are complaining about the time they wait for proper counselling and collection of their medication. Otherwise, we need patients to get services as fast as possible.

P5: I think most of the challenges that are found to be factors which have a negative effect on those HIV-positive women's adherence to HAART have already been mentioned and discussed. For me, there are also some facilitators who help HIV-positive women to take their medication properly. Therefore, as a healthcare provider, what I have understood that things are facilitating adherence in HIV-positive women is the responsibility of HIV-positive women who have children and or families that need care and support from these HIV-positive women. Others like once-daily prescription and less dosage frequency are found to be very helpful for any patient on HAART to be more adherent to her medication.

M: Okay, you have told us the responsibility of the HIV-positive women, prescribe once-daily pills, and reduced dosage frequency are facilitators to HAART adherence of women. Please tell us how these facilitate the adherence of HIV-positive women to HAART.

P10: The adherence to HAART is a very serious issue, especially in women as discussed recently. On the contrary, there are also some other important things which increase HIV-positive women's proper medication-taking practice. As we all know women are very responsible to their children and family. They have a caring nature toward children and other people. They want to help their children and the whole family as long as they are healthy. There are many HIV-positive women who always tell us that they have to take their medication properly daily because they want to be healthy to provide the necessary care to their children as well as to their family members. They know that they will provide the intended care to anyone in need only if they are healthy and thus they take their ARVs as intended to be taken. What I have understood in my day-to-day work in the HAART clinic, there are very careful HIV-positive women in taking of their medication because they want to live longer without ill health due to HIV as they want to provide care for their children, husband and other family members; unless they are healthy, they wouldn’t be responsible to their family.

P4: I know that HIV-positive women have different challenges but still there are some women who want to take their medication properly just to be healthy and then they can provide whatever care to their loved ones including children, relatives, colleagues and old age people. By far they know that this will only be successful if they take their medication properly and thus become healthy and stronger. In our case, the majority of the HIV-positive women are in the age of fertility and had school-age children. They are afraid of dying from HIV/AIDS, but they want to live longer for the sake of their children because if they die their children will be left without parents and become orphans.

P3: Since these HIV-positive women are mothers and their home managers they are highly responsible to their family. Due to that, they think about not only their health but also they think that their family should get the needed support from them [HIV-positive women]. That is why these women take their medication to live longer even for the sake of their children as well as others. The other major thing which I know as a facilitator of adherence to HAART in HIV-positive women is the type of regimen which contains one pill in a day prescription. These days ARVs have been improved in terms of their effectiveness and dosage and frequency. Many HIV-positive women are taking their HAART easily as they intended to take one pill a day and thus, they are very happy with that as this regimen has no pill burden with a one-time taking of a day. These women tell us that they take their medication which means the right dose at the right time on a daily basis because of the easy regimen.

P8: I have seen also many HIV-positive women and even HIV-positive male patients are convenient with single pill preparation of ARVs. It is clear that taking many pills two times a day, especially for a long time, is challenging for our patients; it is one of the reasons for patients have poor HAART adherence. Actually, most of our patients are now taking first-line ARVs and thus they are supposed to take only one pill [fixed single tablet] a day; it is a preparation of two or three drugs combined or fixed in the form of a single tablet. Because of this fixed formulation, the majority of our patients are not raising the issue of ARVs pill burden like that of the situation which was before 4 or 5 years. Most of the patients tell us about the existing simple drug regimen which makes their pills easy to take at the right time.

P4: As my colleagues have said, many HIV-positive women are very responsible to take their medication properly. This is because they want to be healthy and thus they can do whatever they like and desire. In order to have a better health condition, especially in relation to HIV, they know that taking HAART is highly important for their health. I know a couple i.e. husband and wife who always collect their medication on the clinic's scheduled date and take their medication properly. The wife told me that they have 2 children and that she takes care of them, she now has a good health condition because of HAART and thus HAART means life for her.

P2: The other very important issue here is the family support that HIV-positive women have gotten. It is well known that any kind of support which includes psychological, economical, material and the like are very important for HIV-positive patients including those HIV-positive women. Because if HIV positive individual has no kind of support from family, relatives, and others, he/she may not have good attention towards HIV treatment and rather thinks about why you have no support from his beloved ones. This may create psychological stress and other mental issues in her health, therefore, she wouldn't take her medication properly. Atrary, there are women who tell us they take their medication properly as prescribed because they have any kind of support from their family and they become adhere to the medication they are taking. Some HIV-positive women hav also a good look on their health improvement. Because many patients have got tested and diagnosed with HIV after they had been developed different HIV related illnesses due to that they know that how they had suffered a lot. After they have started taking their medication, their health becomes improved, therefore, they are committed to take their medication approperiately.

P1: In my encounter, there are HIV-positive women who come to the clinic to collect their medication and other medical reasons. Yes, I have seen that family support

is highly important for HIV-positive patients, especially for those who are taking their HAART. Because to take their medication at the right time daily, these patients need to have all aspects of support from their close family members. If they get economic and emotional and are reminded of medication-taking support, HIV-positive patients can take their medication properly without fear and discomfort.

P6: By the way the HAART clinic has a strong mechanism of educating their patients. From these, HAART adherence counselling which is provided by all the healthcare providers daily is highly supportive for patients to have a better understanding of HAART and then to take their medication in the appropriate manner. In daily interaction with our patients, we always assess their medication practice just to have an understanding of how they are taking their medication and to know their adherence to their medication subjectively. We ask our patients how they have been taking their ARVs during the last months, weeks and days. Similarly, we assess their challenges while they take their medication if there are any. Based on the information they provide us and the assessment we do, we will have a counselling session together with our patients. The type of counselling that we provide depends upon the individual patient. And thus, what we have understood from our practice, adherence conseling is very helpful to those women patients on HAART to have good adherence to their medication.

P5: As a routine service, we provide counseling on adherence to HAART for every patient. We always have a face-to-face adherence counseling session with our patients. Moreover, we provide our patients as a group with relevant and important messages about their treatment when they stay in the waiting area of the clinic until they refill their ARVs and finalize other personal health issues. This is our day-to-day activity which provides from Monday through Friday.

P2: Adherence counseling on HAART medications is really important for our patients not only for new patients and those with poor adherence to HAART but also for those who are on good adherence just to get them motivated in taking their pills and staying in their good adherence.

P9: In addition to what HIV women patients have got adherence support from the counselling session, they have also gotten information on the benefit of HAART and adherence and life experience from the adherence support peer group which is found in the hospital. Some HIV-positive women tell us that they have improved their medication-taking practice and become more adherent to their medication due to the support they get from this adherence support peer groups. By the way, those HIV-positive women who are members of the adherence

support group of the hospital are all HIV-positive individuals and also they are adherent to their medication. Therefore, their importance is significant because these members provide education about HAART adherence and share their experiences with other HIV-positive women. And this helps those non-adherent women and even those who started their medication newly by having better information and adherence experiences.

P4: On top of the adherence conseling and adherence support which is provided by health care providers and HIV-positive peers, respectively, we are the ones who have a high responsibility to have a standard HIV treatment service within the hospitals. Therefore, we think and work in that direction. HAART clinics should establish sufficient healthcare providers with essential related training. As responsible healthcare providers, we have a good relationship with our patients. We always try to serve patients with respect and a good approach. That is why they are coming and collecting their medication from the HAART clinic always during their clinic schedule.

P7: As it has been said, serving patients with careful attention and a sympathetic approach is an ethical manner which needs to be always practical and given to all patients. We healthcare providers are considering this principle and helping our patients on this ground. But sometimes there are issues which make patients disappointed like waiting a long time at HAART clinics but this also occurred due to acceptable reasons, you know?

M: You are discussing some kind of things which facilitate the medication-taking practice of HIV-positive women; would you add more please if there are others to be mentioned here?

P3: Actually the majority of the things which help HIV-positive women to take their medication properly have been mentioned by these healthcare workers. Just to add a few on this regard, those women who have used a reminder like mobile phone alarms and other electronic devices benefited as these devices remind them to take their medication at the right time while they are at work or sleep and even they forget the medication taking time at all. What I observed in my encounter, those patients who are using any reminder for taking their medication, are more adherent to their HAART than those patients who are not using any kind of reminder for taking their medication. There are one old HIV-positive women who follow her treatment in our clinic and she told me that she always takes her medication when she hears Adhan, Allah ho Akbar, and due to that she doesn't miss her medication...

P9: We understand that those patients taking HAART, females or males, need to get a reminder while in the process of their medication taking. The kind of reminders is different based on the context of the patients. Some of them have family or

other social support which includes just reminding patients to take their medication at the right time for medication taking. Family members like children, husbands and other family members could be mentioned here as they remind HIV-positive family members to take her medication at the right time. Therefore, many women who are taking HAART tell us during their clinic visit day that they have a reminder either electronic reminders like phone and watch alarms or individuals who support them by reminding patients to take medication at the appropriate time. What we observed in this regard, those HIV-positive women who have a reminder of medication taking time, have good experience of taking the HAART at the right time without missing doses and thus they have good adherence to their medication.

P3: When patients [HIV positive women] come to the clinic just to collect their medication, tell us that they take their pills daily as intended without problems because they always use alarms set on their mobile phones and watches.

P7: You have told us about what looks like the experience of HIV-positive women to HAART. From the information you gave us the adherence challenges those women faced are side effects, fear of HIV status disclosure, pill burden, economic problem (lack of money), religious rituals, being busy and forgetting doses some them which prevent those women from taking their medication properly. On the other side, you have also discussed some of the adherence facilitators which help women to take their medication properly and become adherent to their HAART: family support, single pill and easy regimen, women understanding of the benefits of HAART, and adherence support groups.

Do you have any additional ideas or information to be added? Anything you want to add and make it clear?

We have finished our FGDs. I thank you very much for taking the time to participate and for the valuable discussion you made.

END OF INTERVIEW-97 minutes transcribed
